# Cost-effectiveness evidence of mental health prevention and promotion interventions: A systematic review of economic evaluations

**DOI:** 10.1371/journal.pmed.1003606

**Published:** 2021-05-11

**Authors:** Long Khanh-Dao Le, Adrian Cuevas Esturas, Cathrine Mihalopoulos, Oxana Chiotelis, Jessica Bucholc, Mary Lou Chatterton, Lidia Engel

**Affiliations:** Deakin University, Deakin Health Economics, Institute for Health Transformation, School of Health and Social Development, Geelong, Australia; Harvard Medical School, UNITED STATES

## Abstract

**Background:**

The prevention of mental disorders and promotion of mental health and well-being are growing fields. Whether mental health promotion and prevention interventions provide value for money in children, adolescents, adults, and older adults is unclear. The aim of the current study is to update 2 existing reviews of cost-effectiveness studies in this field in order to determine whether such interventions are cost-effective.

**Methods and findings:**

Electronic databases (including MEDLINE, PsycINFO, CINAHL, and EconLit through EBSCO and Embase) were searched for published cost-effectiveness studies of prevention of mental disorders and promotion of mental health and well-being from 2008 to 2020. The quality of studies was assessed using the Quality of Health Economic Studies Instrument (QHES). The protocol was registered with PROSPERO (# CRD42019127778). The primary outcomes were incremental cost-effectiveness ratio (ICER) or return on investment (ROI) ratio across all studies.

A total of 65 studies met the inclusion criteria of a full economic evaluation, of which, 23 targeted children and adolescents, 35 targeted adults, while the remaining targeted older adults. A large number of studies focused on prevention of depression and/or anxiety disorders, followed by promotion of mental health and well-being and other mental disorders. Although there was high heterogeneity in terms of the design among included economic evaluations, most studies consistently found that interventions for mental health prevention and promotion were cost-effective or cost saving. The review found that targeted prevention was likely to be cost-effective compared to universal prevention. Screening plus psychological interventions (e.g., cognitive behavioural therapy [CBT]) at school were the most cost-effective interventions for prevention of mental disorders in children and adolescents, while parenting interventions and workplace interventions had good evidence in mental health promotion. There is inconclusive evidence for preventive interventions for mental disorders or mental health promotion in older adults. While studies were of general high quality, there was limited evidence available from low- and middle-income countries.

The review was limited to studies where mental health was the primary outcome and may have missed general health promoting strategies that could also prevent mental disorder or promote mental health. Some ROI studies might not be included given that these studies are commonly published in grey literature rather than in the academic literature.

**Conclusions:**

Our review found a significant growth of economic evaluations in prevention of mental disorders or promotion of mental health and well-being over the last 10 years. Although several interventions for mental health prevention and promotion provide good value for money, the varied quality as well as methodologies used in economic evaluations limit the generalisability of conclusions about cost-effectiveness. However, the finding that the majority of studies especially in children, adolescents, and adults demonstrated good value for money is promising. Research on cost-effectiveness in low-middle income settings is required.

**Trial registration:**

PROSPERO registration number: CRD42019127778.

## Abstract 2

In a systematic review, Long Khanh-Dao Le and colleagues investigate the cost effectiveness of mental health interventions among children, adolescents, and adults.

## Author summary

**Why was this study done?:**

- Mental health disorders have substantial health and productivity impacts, associated with significant cost to families and the affected individual.
- Promoting positive mental health and preventing mental illness have become key priority goals across various jurisdictions.
- The current review will answer a critical question whether health promotion and prevention interventions provide value for money in children, adolescents, adults, and older adults.

**What did the researchers do and find?:**

- This review summarised evidence on the cost-effectiveness of mental health promotion and prevention interventions from 2008 onwards.
- We identified 65 unique studies, of which, 23 targeted children and adolescents, 35 targeted adults, while the remaining targeted older adults.
- In children and adolescents, screening plus psychological interventions at school were identified as the most cost-effective interventions for prevention of mental disorders, while parenting interventions had good evidence for mental health promotion. In adults, strong evidence supported screening plus psychological interventions for mental disorder prevention, while workplace interventions targeting employees in general were cost-effective. There is inconclusive evidence for preventive interventions for mental disorders or mental health promotion in older adults.

**What do these findings mean?:**

- The majority of the studies consistently found that interventions for mental health prevention and promotion were cost-effective or cost saving. The review found that targeted prevention was likely to be cost-effective compared to universal prevention.
- While studies were of general high quality, there was limited evidence available from low- and middle-income countries, and additional cost-effectiveness studies from these settings are needed.
- Findings of this review should be considered in light of the fact that return on investment (ROI) reports published by governmental agencies are often not published in peer-reviewed literature and thus, may not have been included, but also have the potential to inform on this question.
- The large-scale implementation of mental health promotion and prevention interventions requires consideration beyond cost-effectiveness outcomes.

## Introduction

Mental health is defined as “a state of well-being in which the individual realises his or her own abilities, can cope with the normal stresses of life, can work productively and fruitfully, and is able to make a contribution to his or her community” [1]. Promoting positive mental health and preventing mental illness have become key priority goals across various jurisdictions. The aim of mental health promotion is to increase mental well-being, enhance positive mental health, and empower individuals and communities [2]. Mental illness prevention, on the other hand, focuses on the causes or risk factors of mental illness and aims to reduce the incidence, prevalence, or seriousness of mental health problems, symptoms, and disorders. A commonly used preventive framework in the mental health area was conceptualised by Mrazek and Haggerty, which identified 3 categories of prevention activities: (i) universal (targeting the general population); (ii) selective (targeting high-risk groups); and (iii) indicated (targeting high-risk individuals or groups already displaying symptoms of illness but not meeting full diagnostic criteria) [3]. Apart from high burden of disease [4], mental health disorders have substantial healthcare and productivity impacts, as well as significant cost to families and the affected individual that are viewed as an increasingly recognised economic problem in every country. Mental disorders cost approximately €400 billion in Europe every year [5]. In Australia, around $9.9 billion was spent largely on mental health treatment in 2017 to 2018 [6]. This accounts for 7.6% of the national health spending—while this is not a large proportion of spending compared to the burden of disease associated with mental disorders, it is nonetheless important that this spending constitutes good value for money. One way of determining whether an intervention presents good value for money and desirable use of healthcare resources is through the conduct of an economic evaluation.

Economic evaluations have become an important tool within the priority setting process, whereby decision-makers allocate resources between existing and/or new healthcare services. An economic evaluation is defined as the comparative analysis of interventions in terms of both their costs and their outcomes [7]. There are 4 common types of economic evaluation used in healthcare including cost-minimisation analysis (CMA); cost-effectiveness analysis (CEA); cost-utility analysis (CUA); and cost-benefit analysis (CBA) [7]. CMA refers to the situation where the consequences of 2 or more interventions are “broadly equivalent,” and the differences between them are the costs of these interventions, which is rarely the case in the real world [8]. The distinguishing feature of all other types of economic evaluation is how outcomes (benefits) are measured. CBA measures benefits in monetary terms. More recently, return on investment (ROI) studies have gained interest, which represent a type of CBA by comparing the returns of investing in an intervention with the intervention costs. Compared with CBA, ROIs are limited, as they often do not consider health benefits but only cost offsets within the health sector or other sectors. CEA measures benefits in physical units (e.g., symptom free days), whereas CUA combines both morbidity and mortality into a single unit of measurement, such as a quality-adjusted life year (QALY) gained or a disability-adjusted life year (DALY) averted [7]. CUA analyses are the most frequently used economic evaluation frameworks in international health technology agencies such as National Institute for Health and Clinical Excellence (NICE) in the UK or the Pharmaceutical Benefits Advisory Committee in Australia [9,10].

There have been 2 published reviews of economic evaluations of preventive interventions, focusing on the prevention of mental disorders and the promotion of mental health. Zechmeister and colleagues found 7 studies targeting children and adolescents in a wide range of interventions, such as cognitive behavioural therapy (CBT), peer or crisis support, social work intervention, and early child development programmes that have strong evidence of cost-effectiveness [11]. Mihalopoulos and Chatterton updated that review and found that even though the evidence on the cost-effectiveness of mental health intervention was markedly increasing, there was often a lack of comparability across studies [12]. An important limitation of the review by Mihalopoulos and Chatterton was the focus on preventive interventions for mental disorders and did not include mental health promotion types of interventions as well as ROI studies [12]. Given that there is a growing interest in these types of studies for decision-makers and more economic evaluations have been published since the last review, the aim of this study is to provide an update of the current literature on the cost-effectiveness of mental health promotion and prevention interventions across the age spectrum. The current review will answer a critical question whether health promotion and prevention provide value for money compared to no intervention in children, adolescents, adults, and older adults.

## Methods

This systematic review adheres to the Preferred Reporting Items for Systematic Reviews and Meta-Analyses (PRISMA) guidelines [13] (S1 PRISMA Checklist) and was registered on the PROSPERO database (registration number: CRD42019127778). The review is an update of 2 previously published reviews conducted by Zechmeister and colleagues [11] and Mihalopoulos and Chatterton [12]. This review will summarise evidence on the cost-effectiveness of mental health promotion and prevention interventions from 2008 onwards, taking into account the cutoff time point used in Zechmeister and colleagues [11].

### Identification and selection of studies

An extensive literature search was conducted using electronic databases that included MEDLINE, PsycINFO, CINAHL and EconLit through EBSCO and Embase from January 2008 to October 4, 2020. The search terms used in all these searches were organised into 3 blocks including (i) mental health disorders (e.g., depression, anxiety, and eating disorders [EDs]) and risk factors (e.g., sleep, resilience, and bullying); (ii) promotion and prevention (prevention or promotion); and (iii) economic evaluation (e.g., CEA and CUA). Further details of search terms can be obtained from S1 Table. All citations were imported into an electronic database (Endnote version X8 [14]) in which the duplications were eliminated. A screening web tool system, RAYYAN [15], was then used for the screening process. The retrieved studies were split into 2 groups; each group of references was screened by 2 reviewers (i.e., group 1 screened by ACE and LE; group 2 screened by ACE and LL). A third reviewer from the other allocated group resolved any variation in decisions.

Studies were included if they undertook an economic evaluation or an ROI study (i.e., at least 2 interventions examining both costs and benefits). This excluded partial economic evaluations (i.e., studies that had no comparator or studies that only focused on costs or benefits but not on both). Furthermore, studies related to treatment rather than prevention or promotion were also excluded. The review also focused on studies that only reported mental health conditions (e.g., anxiety disorder), symptoms (e.g., anxiety level), or risk factors (e.g., bullying) as the primary outcome. Studies published before 2008, not in peer-reviewed journal articles, and in languages other than English were excluded. Included studies were categorised according to children and adolescents (aged 0 to 18), adults (aged 18 to 65), and older adults (aged 65 and above). If a study included a mixed population, it was classified based on the mean age of the population included in the study. Studies were categorised into “prevention,” which assessed the cost-effectiveness of an intervention that aimed to reduce the incidence, prevalence, or seriousness of mental health problems and illness, while mental health “promotion” comprised studies that examined the cost-effectiveness of interventions that focused on increasing mental well-being, enhancing positive mental health, and empowering individuals and communities.

### Data extraction

Characteristics of the studies were extracted into a standardised table that was adapted from previous reviews of economic evaluations and the review guideline for economic evaluations developed by the Joanna Briggs Institute [12,16–18]. The data extraction table included characteristics of the population, country, perspective, type of prevention (universal, selective, and indicated prevention), time horizon, type of economic evaluation (i.e., CUA, CEA, CBA, or ROI), study design (i.e., modelled or randomised controlled trial), outcome measures (e.g., QALYs, life years saved, incidence, or severity of clinical outcomes), and cost-effectiveness results (the incremental cost-effectiveness ratio [ICER] or ROI ratio). To make a relevant comparison of the ICERs across the identified studies, all costs were converted into 2020 US dollars. The CCEMG–EPPI-Centre Cost Converter version 1.4 that uses the purchasing power parity approach sourced from the IMF World Economic Outlook database was used to convert all non-US dollar currencies to US dollar currencies [19]. For studies that did not report the reference year, an assumption of 2 years prior to the publication date was made as the base year. Data extraction was undertaken by ACE and OC and double-checked by LL and LE. Disagreements were resolved by discussion between 2 review authors (LL and LE).

### Data synthesis

Economic findings were synthesised and presented as a narrative summary in conjunction with a tabular summary. Given that there is high heterogeneity in terms of population, intervention, comparator, and outcome as well as economic evaluation frameworks across included studies, a meta-analysis was not conducted. Instead, the dominance ranking framework (or permutation matrix) presenting the distribution of studies across 9 possible outcomes in terms of costs and effectiveness was adapted from the systematic review of economic evaluation guidelines developed by the Joanna Briggs Institute [18]. In the dominance raking framework, colour coding was used to indicate implications for decision-makers. A “red” coding shows the situation in which a decision is less favoured or rejected by decision-makers (i.e., costs are higher, and the intervention is less effective). A “green” code indicates the case in which the intervention is strongly favoured (i.e., has better health outcomes and lower costs). A “yellow” coding shows that there is no obvious decision that the intervention is more effective and more costly or less effective and less costly). That is, some form of financial or clinical trade-off is required or a value for money threshold to determine whether the intervention is cost-effective. In cases where an economic evaluation evaluates 2 or more interventions compared to a control, results for each intervention versus no intervention or wait list control was reported separately in the dominance framework table. Similarly, if the study reported results by different perspectives or for different outcome measures, results were reported separately and were ranked “unclear” if the results were conflicting.

### Quality assessment

The Quality of Health Economic Studies Instrument (QHES) was used to assess the quality of included studies [20]. The checklist consists of 16 questions, to be answered with yes or no, and each question is weighted based on importance. Given the lack of a “not applicable” option in the original QHES, we decided that if a question from the QHES was not applicable for a particular study (e.g., the study was a trial-based economic evaluation while the question was related to modelled evaluations), this question was answered with “yes.” Regarding the inclusion of 2 or 3 questions in one assessment criterion in the QHES, studies that partly met a criterion did not achieve a score. The quality score was calculated by adding up all of the points for the questions answered “yes.” Cutoff points were used to determine the quality: 0 to 24 (extremely poor quality); 25 to 49 (poor quality); 50 to 74 (fair quality); and 75 to 100 (high quality). Quality assessment was undertaken by ACE and OC and double-checked by LL and LE. Disagreements were resolved by discussion between 2 review authors (LL and LE).

## Results

**Fig 1:**
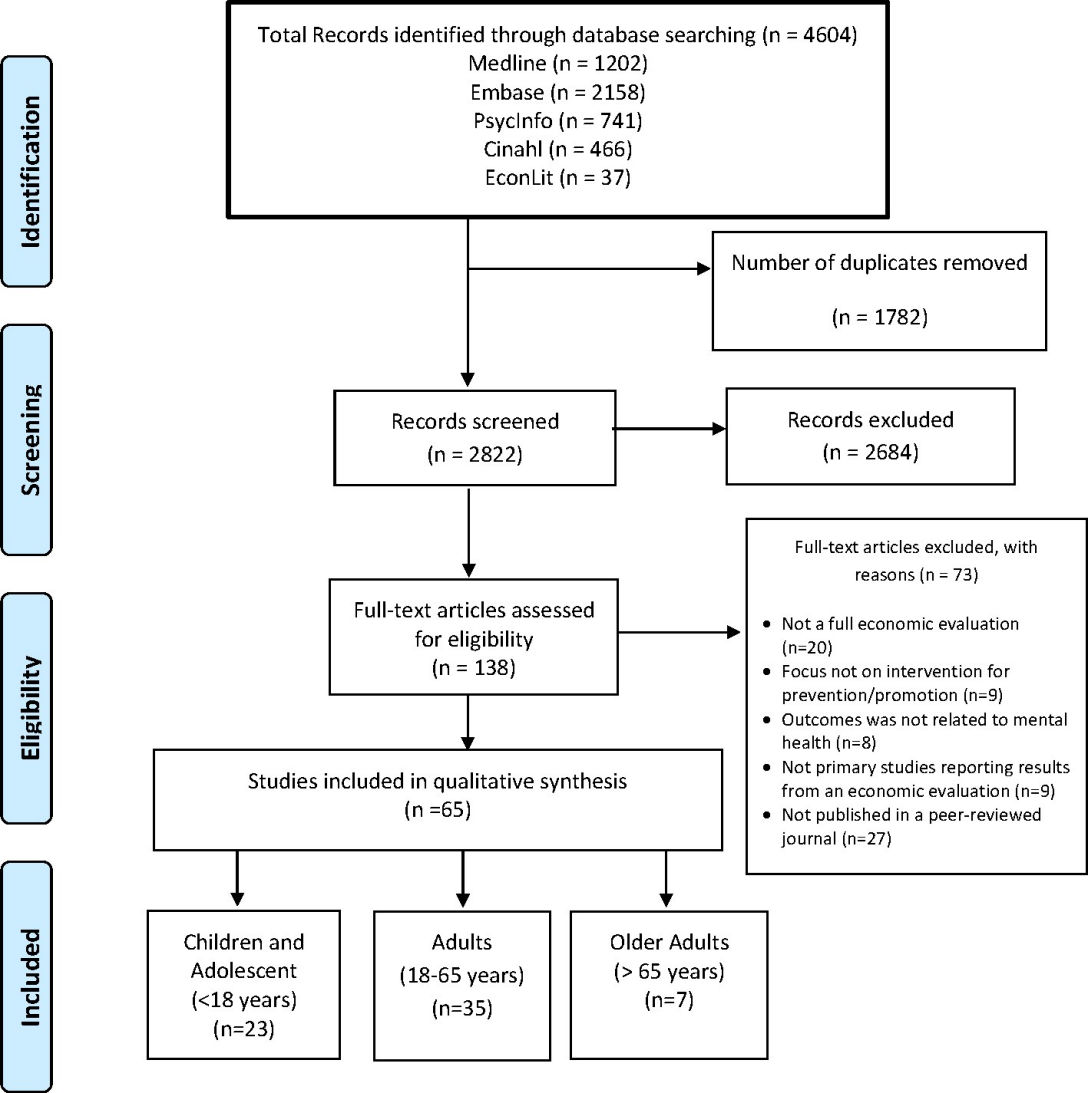
PRISMA flow diagram. PRISMA, Preferred Reporting Items for Systematic Reviews and Meta-Analyses.

The literature search identified 4,604 articles. After excluding duplicate studies, 2,822 studies remained for title and abstract screening. The screening based on title and abstract resulted in 138 eligible studies for full-text screening. Most studies were then excluded because they did not meet the “full economic evaluation” criterion, were not primary studies reporting results of an economic evaluation (e.g., reviews), or focused on treatment rather than prevention or promotion. After the full-text screening, 65 studies were included for data extraction and quality assessment. Further details are presented in the PRISMA flow diagram (Fig 1).

### Characteristics and main findings of included studies

#### Children and adolescents (<18 years)

Table 1 presents the characteristics and main findings of studies focused on children and adolescents. There were 23 studies that evaluated the cost-effectiveness of 33 active interventions for mental health prevention and promotion versus no intervention or wait list control in those aged under 18 years. Studies were conducted in the following countries: US (*n* = 6), Australia (*n* = 7), Sweden (*n* = 5), UK (*n* = 2), the Netherlands (*n* = 2), and multinational (*n* = 1). The most common primary method of economic evaluation used was CEA (*n* = 7) followed by CUA (*n* = 7) and ROI (*n* = 3). Six studies conducted multiple evaluations (CEA + CUA). Most studies (*n* = 12) adopted a societal perspective and a time horizon of 1 year (*n* = 5) or 2 years (*n* = 5), with only 1 study using a time horizon of less than a year. Half of the studies focused on preventive interventions for depression and/or anxiety disorders, followed by mental health promotion (4 studies), suicide (4 studies), EDs (2 studies), and cannabis use (1 study).

**Table 1:** Characteristics of studies included—children and adolescents.

Lead author (year), country	Targeted mental risk factor or condition/disorder	Population description (universal, targeted)	Intervention(s) and comparator (setting, individual or group-based, parenting)	Evaluation type	Study design (trial (*n* =) or model)	Perspective, time horizon	Year of pricing, discount rates	Cost categories	Outcomes	Results ICERs (in 2020 US$ value)^a^	Limitations	Quality score^b^
Anderson and colleagues (2014) [26], UK	Depression	Adolescents aged 12–16 (universal)	CBT (school-based Resourceful Adolescent Program) Usual Care	CEA CUA	Cluster-RCT (*n* = 3,357)	Health and social care sector perspective, 12 months	2012, no discount	Intervention costs and healthcare cost savings	QALYs Symptoms of depression	CBT was less effective and more costly than usual care	CBT designed for adolescents aged 12–15 years Participants were not blinded	86
Lee and colleagues (2016) [23], Australia	Depression	Children and adolescents aged 11–17 (universal and targeted)	Psychological intervention (school-based delivered to groups online or face to face) Do nothing	CUA	Model	Health and education sector perspective, 10 years	2013, 3%	Intervention costs and healthcare cost savings	DALYs	ICER = A$7,350 ($5,592) per DALY averted (universal) and ICER = A$19,550 ($14,875) per DALY averted (indicated)	The paucity of information on the efficacy and cost of the intervention pathways Consider health benefits link to incidence of depression only	100
Mihalopoulos and colleagues (2012) [24], Australia	Depression	Adolescents aged 11–17 (targeted)	Psychological intervention Do nothing	CUA	Model	Health sector perspective, 5 years	2003, 3%	Intervention costs and cost savings	DALYs	ICER = $5,400 ($5,888) per DALY averted	Parameters for adult depression were used. Implementation issues: feasible, notably workforce, financing issues, and acceptability to key stakeholders	87
Philipsson and colleagues (2013) [95], Sweden	Depression Anxiety	Adolescent girls aged 13–18 with internalising problems (targeted)	Physical intervention (dance) Usual care	CUA	RCT (*n* = 112)	Societal perspective, 20 months	2011, 3%	Intervention costs and cost savings	QALYs	ICER = US$3,830 ($4,515) per QALY gained	QoL differs between 2 groups at baseline	100
Stallard and colleagues (2013) [27], UK	Depression	Adolescents aged 12–16 years with high-risk of depression (targeted)	CBT (classroom based) Usual curriculum	CEA CUA	Cluster-RCT (*n* = 5,030)	Societal perspective, 12 months	2010, no discount	Intervention costs, healthcare cost savings	Symptoms of depression (SMFQ) QALYs	CBT was less effective (symptoms of depression) and more costly than usual curriculum CUA ICER = £185,338 ($317,398) per QALY gained	Real-world implementation issues Participants were not blinded No clinical or diagnostic interviews were used	100
Lynch and colleagues (2019) [28], US	Depression	Youths, aged 13–17 with subsyndromal depressive symptoms (targeted)	CBP Usual care	CEA CUA	RCT (*n* = 316)	Health and public service perspective; 2 years (33 months post = randomisation)	Not stated (assumed 2017), 3%	Intervention costs, healthcare costs, family costs (parents time cost), school services, and juvenile costs	DFDs QALYs	ICER = $12,787 ($13,586) per QALY ICER = $14 ($15) per $DFD	Use of indirect methods for translating DFDs into QALYs; 30% missing data; workplace outcomes not considered	93
Ssegonja and colleagues (2020) [25], Sweden	Depression	Adolescents at a start age of 15 years with subsyndromal depression (targeted)	GB-CBT compared with “no intervention”	CEA CUA	Model	Healthcare and limited societal perspective, 5 and 10 years	2018 (US$), 3% for costs and effects	Intervention costs, direct healthcare costs, indirect costs	Cases of depression prevented, QALYs	GB-CBT is dominant (less costly and more effective)	Assumed a constant annual rate of decay of the treatment effect over time; biases in studies from which input parameters were sourced; spillover effects and side effects not considered	100
Mihalopoulos and colleagues (2015) [22], Australia	Anxiety	Inhibited children aged 3–4 (targeted)	Psychoeducational programme (parent focused) Do nothing	CUA	Model	Health sector perspective, 3 years	2013, 3%	Intervention costs and cost savings	DALYs	ICER = $8,000 ($6,151) per DALYs (averted)	Parameters for adults were used; acceptability issues of the intervention to preschools, psychologists as well as the end-users of the intervention	92
Simon and colleagues (2012) [96], the Netherlands	Anxiety	High anxious children aged 8–12 (targeted)	Child-focused CBT Parent-focused CBT Nonintervention	CEA	RCT (*n* = 139)	Societal perspective, 2 years	2008, 4% for costs	Intervention costs, healthcare cost savings	Proportion of ADIS improved children	ICER Child focused vs. no intervention: €2,987 ($4,257) per “ADIS improved” child Parent focused vs. no intervention: cost saving (less costly and more effective)	The ADIS might not be appropriate in preventive context Lower educated mothers dropped out Professional training cost was not included	100
Simon and colleagues (2013) [21], the Netherlands	Anxiety	High anxious children aged 8–12 (targeted)	Child-focused CBT Parent-focused CBT Parent OR child-focused CBT Nonintervention	CEA	Model	Societal perspective, 2 years	2012, 4% for costs	Intervention costs, healthcare costs savings	Proportion of ADIS improved children	Child or parent-focused CBT (based on parental anxiety) was identified as the most cost-effective intervention, with an ICER of €107 ($147) per “ADIS improved” child compared to no intervention	No probabilistic analysis 2-week cost data to extrapolate 1-year cost	100
Chatterton and colleagues (2020) [84], Australia	Internalising problems	Inhibited 4 year olds (targeted)	Screening + parenting program (Cool Little Kids) Usual care	CEA CUA	RCT (*n* = 545)	Societal and health sector, 1 year	2012/2013, no discount	Intervention costs, child’s and parent’s direct healthcare costs; childcare, parental productivity costs	Internalising symptoms (SDQ)-emotional, QALYs	Societal perspective: intervention is cost-effective (dominant); cost per QALY gained: AU$475,213 ($369,843) Healthcare sector: ICER: AU$1,171/SDQ-emotional symptom decrease ($911); cost per QALY gained: AU$130,373 ($101,465)	QALYs based on parental utility; short follow-up; long recall period of 12 months in the resource use questionnaire	88
Ahern and colleagues (2018) [32], Europe	Suicide	Adolescents between 11 and 17 (universal + targeted)	Universal CBT (school based) Indicated school based Screening intervention Do nothing	CEA CUA	RCT (*n* = 11,110)	Payer’s perspective, 1 year	2010, no discount	Intervention costs	Incident suicide attempt **AND** Incident severe suicidal ideation with suicide plans **AND** QALYs	Universal CBT > Screening > Indicated school based. CEA: ICER ($ per 1% point reduction in incident of suicide attempt) Universal CBT vs. control: €35 ($56) Indicated school based: €90 ($137) Screening: €52 ($79) CUA: ICER ($ per QALY gained): Universal CBT vs. control: €47,017 ($75,524) Indicated school based: €120,567 ($182,933) Screening: €64,050 ($97,181)	Mapping utility from SDQ to CHU-9D Cost and outcome data was pooled from 10 European countries and did not reflect country variation level	92
Kinchin and colleagues (2020) [31], Australia	Suicide	Adolescent (15–16 years) (universal)	SafeTALK (3-hour education session) Status quo	ROI	Model	Health and justice systems Societal, 5 years	2014, 5%	Intervention costs, healthcare and non-healthcare saving	Cost saving	Health and justice: Mackay: ROI = 1.45 Queensland ROI = 0.19 Australia ROI = 0.15 Societal: Mackay: ROI = 31.2 Queensland ROI = 4.1 Australia ROI = 3.3	Effectiveness sourced from Signs of Suicide programme Did not account reattempted suicide Coronial inquiry, police, and ambulance costs were sourced from literature Probabilistic sensitivity analysis was not preformed	90
Godoy Garraza and colleagues (2018) [30], US	Suicide	Youth aged 16–23 (targeted)	Multicomponent programme (The GLS Suicide Prevention Program) Do nothing	ROI	Model	Health sector perspective, 3 years	2010, 3%	Intervention costs and healthcare cost saving	Cost saving	Estimated benefit–cost ratio = 5	Reduction in suicide did not source from RCT. Averted health expenditures were derived from secondary sources, rather than health cost data collected in the context of the programme	89
Gray and colleagues (2011) [29], US	Suicide	Males aged 13–16 in Utah’s Third District Juvenile Court who had 2 to 12 offenses (targeted)	Best practice: early mental health intervention vs. control group	CEA	Matched-control study (*n* = 719)	Not reported (probably health sector), 1 year	Not stated	Observation and assessment costs, detention cost, youth corrections and treatment costs	Recidivism, days in court placement, and The Youth Outcome Questionnaire scores	Cost saving (less costly and more effective in improvement of the Youth Outcome Questionnaire scores)	Non-RCT Low participation rate Under-presented sample	78
Le and colleagues (2017) [33], Australia	Anorexia nervosa and bulimia nervosa	Adolescent aged 15–18 years old; secondary school girls with high body image concerns (targeted)	Cognitive dissonance (school based) Do nothing	CUA	Model	Societal perspective, 10 years	2013, 3%	Intervention costs and healthcare cost savings	DALYs	ICER = A$103,980 ($76,356) per DALY averted	Included only 2 types of EDs Crossover rates were not addressed in the model Low participation rate	100
Wang and colleagues (2011) [34], US	Bulimia nervosa	Adolescence girls aged 13.5 (universal)	School-based education + physical activity Usual curricula	CUA	Model	Societal perspective, 10 years	2010, 3%	Intervention costs, medical costs saved	QALYs	ICER = $9,751 ($11,887) per QALYs combined prevention of obesity and DWCB (own calculation)	Medical costs for the treatment of subthreshold BN or travel costs related to treatment of BN were not included Long-term medical cost estimate and the HRQL estimate were based on a single study	65
Beckman and Svensson (2015) [35], Sweden	Bullying	Adolescents (universal)	Whole-school approach	CEA	Model	Public payer perspective, 3 years	2014, 3%	Intervention costs	Bullying victim spared	ICER = 131,250 Swedish krona ($16,744) per victim spared	Efficacy of intervention was based on a small quasi-experimental study	93
Deogan and colleagues (2015) [39], Sweden	Cannabis use	Adolescents aged 14 or 15; eighth grade of compulsory school, (universal)	ALERT plus ATOD Ordinary ATOD only	CUA	Model	Societal perspective, 20 years	2013, 3%	Cost of interventions and healthcare and non-healthcare cost saving	QALYs	ICER = €22,384 ($30,407) per QALY within the Swedish context or cost saving within the US context	Results were sensitive to the follow-up period, effectiveness, and prevalence of cannabis use	87
Ocasio and colleagues (2014) [38], US	Challenging behaviour	Three- and 4-year old children enrolled in 4 urban preschools in northern New Jersey (universal)	Tiered approach: Second Step curriculum, mental health clinician and play therapy	CEA	Pre-post study (*n* = 268)	Unclear (education sector), 2 years	2012, not stated	Cost of intervention	The PKSB-2	The results indicated that the $900 ($1,024) per child cost resulted in increasing behaviour scale scores around 10% to 15% compared to the baseline scores	No comparator Small sample size Inconsistency in the data collection methods	65
Nystrand and colleagues (2020) [97], Sweden	Externalising problems (attention deficit/hyperactivity problems and conduct problems)	Parents of children aged 5–12 (targeted)	Five indicated parenting interventions (4 group-based and 1 self-help book) Wait list control	ROI	Model	Payer perspective, 2 years and long-term productivity	2015, 3% for costs and outcomes	Intervention costs, healthcare and educational sector costs, productivity costs	Cost saving	ROIs (trial): Comet: 7 Connect: 10.61 Incredible years: 5.96 COPE: 15.80 Self-help book: 328.04	Cost data not collected alongside the trial but estimates from the literature; parents’ health and well-being not considered	94
Dalziel and colleagues (2015) [37], Australia	Maltreatment	Children with opioid-dependent parents (targeted)	PuP programme Combination of “Usual Care” and “Brief Intervention” groups	CEA	RCT (*n* = 64)	Societal perspective, 6 months	2013, no discount	Screening and enrolling cost, PuP cost 7 comparison interventions costs	Case of maltreatment measured by CAP inventory score	ICER = A$43,975 ($33,936) per case of maltreatment avoided	Detection maltreatment issues	93
Herman and colleagues (2015) [36], US	Mental health (in women and their children)	Divorced women with at least 1 cohabitating child between 9 and 12 years old (targeted)	Parenting-focused programme Parenting-focused programme with child focus No intervention	CBA	RCT (*n* = 202 mothers and 194 adolescents)	Societal, 15 years	2007, 3%	Intervention costs, healthcare and non-healthcare cost saving	No clinical outcomes were measured	Cost saving (i.e., the intervention was associated with cost saving from healthcare and criminal justice that offset the intervention cost)	Adherence intervention issues. Under-presented sample	81

^a^CCEMG-EPPI-Centre Cost Converter: web-based tool for adjusting estimates of cost on November 2, 2020 using IMF source dataset for PPP.

^b^Quality assessment was undertaken using the QHES checklist.

ADIS, Anxiety Disorder Interview Schedule; ALERT, Adolescent, learning, Experiences, Resistance, and Training; ATOD, Alcohol, Tobacco, and Other Drug; BN, Bulimia Nervosa; CAP, child abuse potential; CBA, cost benefit analysis; CBP, cognitive-behavioural depression prevention program; CBT, cognitive behavioural therapy; CEA, cost-effectiveness analysis; CHU9D, Child Health Utility 9D; CUA, cost-utility analysis; DALY, disability-adjusted life year; DFD, depression-free day; DWCB, disordered weight control behavior; GB-CBT, group-based cognitive behavioural therapy; HRQL, health-related quality of life; ICER, incremental cost-effectiveness ratio; PKSB-2, Preschool and Kindergarten Behavior Scales-Second Edition; PPP, purchasing power parity; PuP, Parents under Pressure; QALY, quality-adjusted life year; QHES, Quality of Health Economic Studies Instrument; QoL, quality of life; RCT, randomised controlled trial; ROI, return on investment; SDQ, Strengths and Difficulties Questionnaire; SMFQ, Short Mood and Feelings Questionnaire.

The economic evidence of psychological interventions targeting high-risk populations for prevention of anxiety disorders consistently reported that child-focused CBT, parent-focused CBT, or parent-focused psychoeducational interventions provided good value for money. Importantly, a modelled economic evaluation by Simon and colleagues [21] found that offering child- or parent-focused interventions based on parental anxiety were cost saving compared to offering child- or parent-focused interventions to all parents under a societal perspective. Within the Australian context, Mihalopoulos and colleagues [22] and Chatterton and colleagues found that a parent-focused intervention (Cool Little Kids) was cost-effective (i.e., falling well below the specified value for money threshold) for indicated prevention of anxiety disorders or internalising problems.

Economic evidence for the prevention of major depressive disorder (MDD) is more controversial. Three modelled economic evaluations (using pooled evidence of effectiveness where possible) showed that school-based psychological interventions (e.g., CBT) were cost-effective regardless of preventive strategies (universal or indicated) compared to no intervention [23–25]. These studies used a 10-year time horizon and considered costs related to health and non-health sectors such as productivity costs or costs to the education sector. In contrast, 2 trial-based economic evaluations found that school-based CBT was not cost-effective—in fact, more costly and less effective than usual care for indicated prevention of MDD with a 1-year follow-up [26, 27]. Another trial-based evaluation conducted by Lynch and colleagues [28] found that CBT delivered to adolescents with subsyndromal depressive symptoms in community settings was more effective and more costly with the ICER of US$13,586 per QALY.

For the prevention of suicide, a multicomponent programme (combined gatekeeper training, promotion of national suicide prevention hotlines, and education and awareness activities) demonstrated cost savings within the US context [29,30]. Within the Australian context, a suicide awareness training (i.e., a 3-hour education session) delivered to secondary school students aged 15 to 16 was found to be cost saving with an ROI ratio of 3.28 under a societal perspective but more effective and more costly under a health sector perspective [31]. In contrast, a universal intervention indicated that CBT and a screening intervention plus treatment or healthy lifestyle programme for high risk of suicide at school were found not to be cost-effective compared to educational posters within the UK context [32].

There were 2 studies that investigated the cost-effectiveness of preventive interventions for EDs. Within the Australian context, Le and colleagues found that a cognitive dissonance intervention targeting females with high body image concerns was not cost-effective for the prevention of anorexia nervosa and bulimia nervosa [33]. However, the authors noted that the intervention became cost-effective if 90% of eligible students (i.e., females with high body image concerns) agreed to participate in the intervention [33]. Universal school-based obesity prevention programmes were cost-effective for the prevention of bulimia nervosa and even cost saving if the obesity prevention benefits were also included [34].

Regarding mental health promotion, preventive interventions for bullying have demonstrated good value for money with an ICER of KR$131,250 (or $16,744) per QALY [35]. A parenting-after-divorce programme targeting both mothers and their children evaluated in a RCT was cost saving; it demonstrated a reduction in mental health costs and justice system service use during a follow-up time of 15 years [36]. The cost-effectiveness of other interventions could not be determined due to the absence of a willingness-to-pay threshold for clinical outcomes (e.g., behaviour scores and cases of maltreatment avoided) used in these studies [37,38]. A modelled evaluation found that a school-based intervention for prevention of cannabis use might be cost saving in the US context but not in the Swedish context [39]. A parenting programme targeting divorced women, with or without an additional child focus, was also cost saving given that it improved mental health and well-being in both parents and their children [36].

#### Adults (18 to 65 years)

There were 33 economic evaluations (from 35 publications) of mental health promotion and prevention interventions targeting adults; 2 studies reported follow-up findings in separate publications [40,41] (see Table 2). Half of these studies (*n* = 16) evaluated interventions conducted in 6 European countries (the UK, the Netherlands, Norway, Belgium, Spain, and Germany) followed by the US (*n* = 9), Canada (*n* = 4), Australia (*n* = 2), Sri Lanka (*n* = 1), and Japan (*n* = 1). The majority of economic evaluations applied conventional economic techniques such as CEA, CUA, and CBA (*n* = 25). Six studies conducted multiple evaluation frameworks (e.g., CEA and CUA) and 2 conducted an ROI. Most studies were conducted from healthcare perspective only (*n* = 11), followed by a societal perspective (*n* = 9) or other perspectives (*n* = 9). Three studies were adopted both societal and health sector perspective. The most common time horizon used in the included studies was up to 1 year (*n* = 18), ranging from a minimum time horizon of 12 weeks to 1-year time horizon. A total of 15 studies adopted a time horizon that was longer than 1 year. Nearly a third of the studies (11/35 studies) focused on prevention of depression or MDD. Of the remaining studies, 8 studies focused on suicide prevention [42–49] 7 focused on mental health and well-being [40,50–55], 2 focused on prevention of EDs [56, 57] or prevention of psychosis [41,58], and 3 focused on prevention of substance use [59], anxiety disorder [60], or panic disorder [61].

**Table 2:** Characteristics of studies included—adults.

Lead author (year), country	Targeted mental risk factor or condition/disorder	Population description (universal, selective, indicated)	Intervention(s) and comparator (setting, individual or group-based, parenting)	Evaluation type	Study design (trial (*n* =) or model)	Perspective, time horizon	Year of pricing, discount rates	Cost categories	Outcomes	Results ICERs (in 2015 US value)^a^	Limitations	Quality score^b^
Kumar and colleagues (2018) [60], US	Generalised anxiety disorder	Persons with no or mild anxiety (universal)	Mobile CBT compared to traditional face-to-face CBT or no CBT	CUA	Model	Societal and payer perspective, lifetime	2016 US$, 3% for costs and QALYs	Intervention cost, medical care, pharmaceutical, costs associated with disability days	QALYs	ICER: dominant (i.e., less costly and more effective) (for both comparators)	Effectiveness of pharmacotherapy in combination with CBT not factored and assumed that persons are on pharmacotherapy for their entire life; effectiveness data sourced from a small pilot study	84
Lintvedt and colleagues (2013) [69], Norway	Depression	Unclear	e-CBT (MoodGYM and BluePages) No intervention	CUA ROI	Model	Unclear, 1 year	2009, no discount	Intervention costs	QALYs Cost saving	ICER = NOK$3,432 ($506) per QALY ROI = 9	Short-term efficacy used to extrapolate long-term effectiveness Completer analysis	35
Dukhovny (2013) and colleagues [71], Canada	Depression	Women with a high risk of PPD (targeted)	Peer support intervention Usual care	CEA	RCT (*n* = 610)	Societal prospective, 12 weeks	2011, not applicable	Public health cost, volunteer opportunity cost, hired housework, hired childcare, family/friend time off work, healthcare utilisation, inpatient admissions	Case of PPD averted	ICER = C$10,009 ($9,415) per case of PPD averted	Generalisability issues ICER is sensitive to programme cost Short time horizon	100
Henderson and colleagues (2019) [72], UK	Depression	Mothers at low risk of postnatal depression (universal)	PoNDER health visitor training (intervention cluster) Control cluster	CEA CUA	Cluster RCT (*n* = 1,459)	NHS and social care perspective, 6 months	2004, not applicable	Intervention cost, direct healthcare costs	EPDS, QALYS	ICER = dominant (less costly and more effective) ($6,859)	Short time horizon, limited perspective, missing data	93
Lokkerbol and colleagues (2014) [67], the Netherlands	Depression	Adults aged 18–65 years (universal)	e-health psychological self-help interventions + usual care Usual care	ROI	Model	Healthcare, 5 years	Unclear Costs 4% Effects 1.5%	Unclear	DALYs	Cost saving ROI = increase from 1.45 to 1.77	Threshold analysis No sensitivity analysis	66
Mihalopoulos and colleagues (2011) [62], Australia	Depression	Adults with subthreshold depression (targeted)	Brief bibliotherapy Group-based CBT Do nothing	CUA	Model	Health sector, 5 years	2003, 3%	Intervention costs, healthcare cost saving	DALYs	ICER: $9,303 per DALY for brief bibliotherapy and $21,636 for CBT	Acceptability of the intervention Decay rate of the effectiveness of the intervention	100
Buntrock and colleagues (2017) [68], Germany	Depression	Adults with depressive symptoms (CES-D ≥16) (targeted)	iPST/BA + TAU (202) Enhanced TAU (204)	CEA, CUA	RCT (*n* = 406)	Societal Health sectors, 1 year	2013, no discount	Intervention costs	QALYs Depression-free year	ICER: Societal perspective: €13,400 ($19,501) per QALY or €1,117 ($1,626) per depression-free year Healthcare perspective: €13,500 ($19,646) per QALY or €1,125 ($1,637) per depression-free year	Short time horizon Not differentiate between prevention of first-ever onsets of MDDs or MDD recurrences Highly educated samples	100
Hunter and colleagues (2014) [63], UK	Depression	Adult primary care population with no current diagnosis of depression (universal and targeted)	Screening by a risk algorithm plus low-intensity depression prevention intervention Universal low-intensity intervention (online CBT or bibliotherapy) TAU	CUA	Model	Health sector, 1 year	2010–2011, no discount	Intervention costs	QALYs	ICER: Cost saving compared to universal prevention and £9,608 ($16,085) per QALY relative to treatment as usual	Short term time horizon The intervention pathway is well justified given lack of supported evidence Threshold analysis	93
van den Berg and colleagues (2011) [65], the Netherlands	Depression	Adults aged 20–65 visiting GP with subthreshold depression (targeted)	Screening + minimal contact psychotherapy Usual care	CUA	Model	Health sector Societal, 5 years	2008 Costs 4% Effects 1.5%	Intervention costs, healthcare and non-healthcare cost saving	DALYs	Cost saving under societal perspective Cost-effective under health sector perspective ICER: €1,400 ($1,995) per DALY	Short-term (i.e., 1 year) effectiveness of the intervention based on a single trial that was underpowered Disability weight based on a small, unpublished study	100
Fernández and colleagues (2018) [64], Spain	Depression	Adults in primary care (targeted)	Screening by a risk algorithm plus low-intensity depression prevention intervention TAU	CUA	RCT (*n* = 3,326)	Health sector Societal, 18 months	2012, 3.5%	Intervention costs, healthcare and non-healthcare cost saving	QALYs	Societal perspective: ICER–cost saving Health sector perspective: ICER = €1,327 ($2,196) per QALY	Underrepresented sample Unblinded trial	100
Jiao and colleagues (2017) [66], US	Depression	Adults aged 20 years (universal)	Two-stage screening with PHQ-2 and PHQ-9 with collaborative care No screening	CUA	Model	Societal, 50 years	2015, 3%	Intervention costs, healthcare and non-healthcare cost saving	QALYs	ICER = US$1,726 ($1,889) per QALY gained	No account for comorbidity Triangular distribution was used	85
Goetzel and colleagues (2014) [70], US	Modifiable risk factors including depression	Workers in small businesses (universal)	Health risk management programme No intervention	ROI	Pre-post study (*n* = 2,458) + model	Unclear, 1 year	2010, no discount	Intervention costs, healthcare and non-healthcare cost saving	Cost saving	Cost saving ROI = 2.03	Non-RCT design with short term time horizon Under-presented sample Underestimation of programme cost	75
Ising and colleagues (2015, 2017) [73,76], the Netherlands	Psychosis	Adults with ultra-high risk for psychosis (targeted)	CBT+TAU (95) TAU (101)	CEA, CUA	RCT (*n* = 201)	Mental healthcare Societal, 4 years	2014 Costs 4% Effects 1.5%	Intervention costs, costs related to psychiatric healthcare, costs of medication, and participants’ travel costs	Prevented psychosis QALYs	ICER–cost saving (less costly and more QALY gained)	High dropout rate Not capture other medications than antipsychotic medication No control for baseline differences	100
Wijnen and colleagues (2020) [58], the Netherlands	Psychosis	Individuals with ultra-high risk psychosis (targeted)	CBT Care as usual	CUA	Model	Health sector, 10 years	2018 Costs 4% Effects 1.5%	Interventions and healthcare savings	QALYs	Cost saving (less costly and more QALY gained)	Individual patient characteristics were not taken in account The costs of identifying persons at ultra-high risk psychosis were not included	99
Akers and colleagues (2017) [57], US	EDs	Young women (mean age of 21.6 years) with high body image concerns (targeted)	Cognitive dissonance intervention Educational brochure	CEA	RCT (*n* = 408) + model	University, 3 years	2012, no discount	Intervention costs	Cases with clinical meaningful change on the ED symptom scales	ICER: US$838 ($961) per case with clinical meaningful change on the ED scales	No probabilistic analysis Narrow perspective Uncommon outcome	54
Kass and colleagues (2017) [56] [56], US	EDs	Students (universal)	Screening + preventive or treatment interventions Wait list	CEA	Model	Payer, unclear	2016, unclear	Intervention costs, healthcare cost saving	ED cases	Cost saving (i.e., the intervention is associated with less costly and fewer individuals needing in-person psychotherapy than control)	Exclusion of screening cost Underestimation of EDs treatment costs No probabilistic or sensitivity analysis	49
Iijima and colleagues (2013) [50], Japan	Mental health	Employees (universal)	Mental health prevention (unclear) No intervention	CBA, ROI	Cross-sectional survey (*n* = 12,864) + model	Employer, 1 year	Unclear, no discount	Intervention costs, non-healthcare cost saving	Cost saving	Cost saving ROI ratio 1.55	Non-RCT design No probabilistic or sensitivity analysis	28
Murphy and colleagues (2012) [51], UK	Mental health	People aged 16–88 years with CHD or mental health (targeted)	The Wales National Exercise Referral Scheme Usual care or brief written information	CUA	RCT (*n* = 2,160)	Public sector, 1 year	Unclear (2010), no discount	Intervention costs	QALYs	ICER = £12,111 ($20,665) per QALY gained	High dropout rate and short-term time horizon Under-representative sample No probabilistic or sensitivity analysis	83
Müller and colleagues (2019) [55], Germany	Mental health	Adult insurance holders of the German insurance fund AOK (universal)	Mindfulness-based mental health promotion programme “Life Balance” Usual care	CEA	Non-RCT (*n* = 1,166)	Healthcare and societal perspective, 1 year	Unclear (assumed 2017), no discount	Intervention costs, direct costs, indirect costs	Self-reported mental health based on the HADS	Societal perspective ICER dominant (i.e., less costly and more effective) Healthcare perspective: ICER = €91 ($124)	Non-RCT and use of propensity score matching; high non-response rate and drop put rate; not all costs considered in societal perspective; short-term time horizon	100
Noben and colleagues (2014, 2015) [40,74], the Netherlands	Mental health	Nurses with elevated risk of mental health (targeted)	Screen-positive nurses received personalised feedback + occupational physician Screening without feedback + usual care	CBA	RCT (*n* = 617)	Organisation, 6 months	2011, no discount	Intervention costs, non-healthcare cost saving	Cost saving	ROI = 7	High dropout Costs of staff turnover and the spill-over effects were not included Short-time horizon	87
Ride and colleagues (2016) [52], Australia	Mental health	First-time mothers (targeted)	Psychoeducational intervention (What were we thinking) Usual care	CEA, CUA	RCT (*n* = 359)	Public sector, 6 months	2013–2014, no discount	Intervention cost, healthcare and non-healthcare cost saving	QALYs, 30-day prevalence of depression, anxiety, and adjustment disorders	ICER = A$36,158 ($27,679) per QALY gained ICER = A$151 ($116) per reduction in 30-day mental disorder prevalence	Short-term time horizon Considerable uncertainty around ICER	100
Thanh and colleagues (2013) [54], Canada	Mental health	High-risk individuals who were referred to the service network for diagnostic services or those who were diagnosed with FASD and were referred to the service network for support services (targeted)	Service network No service network	CBA	Model	Societal, 1 year	2012, no discount	Intervention costs, healthcare and non-healthcare cost saving	Crime, homelessness, mental health problems, and school disruption (for children) or unemployment (for adults)	Cost saving ROI > = 1 if effectiveness of the programme > = 28%	Threshold analysis (i.e., lack of intervention effectiveness data) Factors such as having reliable differential diagnosis, access to service, compliance with programme, baseline health status, and other influencers were not considered	67
Schotanus-Dijkstra and colleagues (2018) [53], the Netherlands	Mental health	Participants with suboptimal levels of mental well-being (targeted)	An email-guided positive psychology vs. a wait list control group	CEA	RCT (*n* = 275)	Health sector perspective, 6 months	2014, no discount	Intervention costs: direct medical and direct non-medical costs	Flourishing mental health Treatment responders for anxiety and depressive symptoms measured by MHC-SF	ICER = €2,359 ($3,220) for flourishing, €2,959 ($4,039) for anxiety, and €2,578 ($3,519) for depression	Overrepresented well-educated women ICER for each type of mental health symptoms	81
Pil and colleagues (2013) [42], Belgium	Suicide	People at risk for suicide (targeted)	Suicide Helpline No intervention	CUA	Model	Societal, 10 years	2012 Costs 3% Effects 1.5%	Intervention costs, healthcare and non-healthcare cost saving	QALYs	The Suicide Helpline is less costly and produces more QALY gains compared to no intervention	Did not include reattempted suicide. US data rather than local data were used to inform the model	92
Atkins and Woods (2013) [44], US	Suicide	Unclear (universal)	Suicide Barrier on the Golden Gate Bridge No intervention	CBA	Model	Societal, 20 years	Unclear Unclear	Intervention costs	DALYs Value of statistical life	ICER = US$4,876 ($5,700) per DALYs	No uncertainty analysis No description of costing method	22
Lebenbaum and colleagues (2020) [49], Canada	Suicide	Adults age 16+ (universal)	Multicomponent intervention No intervention	CUA	Model	Societal, 50 years	2016, 1.5%	Intervention costs, healthcare and non-healthcare saving	QALYs	ICER = CAD $18,853 ($16,916) per QALY	Did not take into account the high variability of suicide rates among subpopulation Did not include caregiver cost Did not include suicide attempts without hospitalisation	99
Damerow and colleagues (2020) [48], Sri Lanka	Suicide	Unclear (universal)	Shop-based gatekeeper training programme	CEA	Model	Government, 3 years	2019 (US dollars), 3%	Intervention costs	Suicide cases	The programme needs to prevent an estimated 0.23 fatal pesticide self-poisoning cases over 3 years to be considered cost-effective	Threshold analysis Cost data from experts’ opinion Underestimated administrative costs	58
Dunlap and colleagues (2019) [47], US	Suicide	Adults at emergency department (targeted)	Universal screening Universal screening + telephone Usual care	CEA	Controlled study (*n* = 1,376)	Provider, 1 year	2015, no discount	Intervention costs	Suicide and suicide attempts	ICER: Screening: $2,789 ($3,053) per suicide or suicide attempt averted Screening + telephone: $5,020 ($5,494) per suicide or suicide attempt	Some data replied on assumptions from research team Administrative and training costs were excluded in universal screening	82
Haddock and colleagues (2019) [46], UK	Suicide	Adults in acute psychiatric wards (targeted)	Cognitive–behavioural suicide prevention therapy + TAU TAU	CUA	RCT (*n* = 51)	Health and social care, 6 months	2015–2016, no discount	Intervention costs, health and social care service cost	QALYs	Less effective and less costly	Underrepresented sample (i.e., 1 centre only Cross contamination with other treatments Non-statistical significant QALYs and costs	93
Vasiliadis and colleagues (2015) [43], Canada	Suicide	Patients with depression (targeted)	NAD multimodal suicidal prevention programme	CEA	Model	Healthcare and societal, 1 year	2010 3%, 5%	Suicidal prevention programme costs, direct medical and non-direct medical costs, others (police investigation and funeral costs), lost productivity cost	Reduction in suicide attempts (life saved years)	ICER = CAD$3,979 ($3,864) per life year saved	Province-level cost data Pre-post study without a control	83
Denchev and colleagues (2018) [45], US	Suicide attempts	Hospital emergency department patients aged 18+ (targeted)	Follow-up via postcards or caring letters, follow-up via TO, and suicide-focused cognitive-behavioural therapy (CBT)—with usual care.	CEA	Markov model	Not stated (probably health sector), 54 weeks	2014, no discount	Intervention costs, costs associated with the index visit and any subsequent ED visit and with inpatient and outpatient care following ED presentation	Suicide attempts averted, life-years saved	Postcards intervention was dominant (more effective and less costly) to usual care, ICER = $4,300 ($4,757) for TO and $18,800 ($20,797) for CBT compared to usual care	Replying on experts’ opinion Trials to inform effectiveness evidence had lacked power to assess suicide death Low sensitivity of detecting ED patients’ suicide risk that impact on population implementation	90
Miller and colleagues (2007) [59], US	Substance abuse	US transportation company (universal)	Peer-based substance abuse prevention programme	ROI	Retrospective ecological study	Workplace and societal, 2 years	3%, 1999	Injury cost, PeerCare cost, substance abuse testing cost	Cost saving, injury cost avoided per dollar invested and per employee	ROI = 27 ROI = $35 ($53) per employee	Under-presentative sample No comparator	56
Smit and colleagues (2009) [61], the Netherlands	Panic disorder	Adults with panic disorder symptoms not meeting the DSM-IV panic disorder (targeted)	Time-limited CBT vs. care as usual	CEA	RCT (*n* = 117)	Societal perspective, 3 months	2003, no discount	Direct medical and direct non-medical costs.	DSM-IV PD-free survival	ICER = €6,198 ($9,766) per PD-free survival gained	Short-term time horizon Baseline difference Uncertainty about intervention implementation	96

^a^CCEMG-EPPI-Centre Cost Converter: web-based tool for adjusting estimates of cost on November 2, 2020 using IMF source dataset for PPP.

^b^Quality assessment was undertaken using the QHES checklist.

CBA, cost benefit analysis; CBT, cognitive behavioural therapy; CEA, cost-effectiveness analysis; CES-D, Center for Epidemiological Studies Depression Scale; CHD, coronary heart disease; CUA, cost-utility analysis; DALY, disability-adjusted life year; DSM, Diagnostic and Statistical Manual of Mental Disorders; ED, eating disorder; EPDS, Edinburgh Postnatal Depression Scale; FASD, Alberta Fetal Alcohol Spectrum Disorder; GP, general practitioner; HADS, Hospital Anxiety and Depression Scale; ICER, incremental cost-effectiveness ratio; MDD, major depressive disorder; MHC-SF, Mental Health Continuum-Short Form; NAD, Nuremberg Alliance against Depression; NHS, National Health Service; PD, panic disorder; PHQ, Patient Health Questionnaire; PPD, postpartum depression; PPP, purchasing power parity; QALY, quality-adjusted life year; QHES, Quality of Health Economic Studies Instrument; RCT, randomised controlled trial; ROI, return on investment; TO, telephone outreach; VAS, visual analogue scale.

Screening adults at high-risk of MDD with or without provision of minimal contact (e.g., brief CBT or brief psychotherapy) was found to be consistently cost-effective, even cost saving compared to current practice [62–66]. Within the Australian context, Mihalopoulos and colleagues evaluated the modelled cost-effectiveness of a brief bibliotherapy and CBT intervention for adults with subthreshold depression [62]. The study showed that both interventions were cost-effective compared to a “doing nothing” scenario, but brief bibliotherapy was more favourable than CBT, although it had a much wider uncertainty interval [62]. Screening adults for risk of depressive symptoms and providing a low intensity depression preventive intervention was found to be cost-effective compared to universal prevention or doing nothing in selected European countries [63–65]. Jiao and colleagues suggested that a 2-stage depression screening plus early intervention in the US resulted in an ICER of $1,726 (or $1,889) per QALY gained [66]. Internet-based CBT for the prevention of MDD was examined in 2 trial-based evaluations. Both studies consistently suggested that internet-based CBT was not cost-effective compared to treatment as usual [67,68]. Although a favourable result for internet-based CBT was found in Norway, this study actually included both those with subclinical depressive disorder and those with diagnosed depressive disorder. The results were not reported separately [69]. Other interventions included depression as a study outcome [70] or used clinical outcomes, making it difficult to determine whether these interventions were cost-effective [70,71]. A trial-based evaluation found that health visitor training to assess postnatal depression and deliver psychological therapy to women at risk of depression was cost saving within the UK context [72].

A telephone “Helpline” available to adults who are at risk of suicide or constructing a suicide barrier on the Golden Gate Bridge in San Francisco, California in the US were found to be cost saving in terms of prevention of suicide in Belgium or the US, respectively [42,44]. For adults who attended hospital emergency department due to self-harm, distributing postcards providing messages of psychosocial support to individuals after discharge was found to be cost saving, while telephone outreach and CBT were more effective and more costly for prevention of suicide attempts [45,47]. A multicomponent suicide prevention programme targeting adults with depression was consistently found to be more effective and more costly with an ICER below the common threshold of $50,000 per QALY in 2 modelled evaluations within the Canadian context [43,49]. Delivering CBT to in-patients in acute psychiatric wards was questionable for suicide prevention given that the intervention was found to be less effective and less costly. A threshold analysis by Damerow and colleagues [48] indicated that a shop-based gatekeeper training programme would be cost-effective if it was able to prevent an estimated 0.23 fatal pesticide self-poisoning cases over 3 years within the Ski Lankan context.

One study investigating the cost-effectiveness of a cognitive dissonance intervention for the prevention of EDs targeting female university students with high body image concerns reported an ICER of US$856 (or $961) per additional at-risk person reducing ED symptoms [73]. Another study showed that a stepped care model for online prevention and treatment among US college students was cost saving [56]. For adults at ultra-high risk for psychosis, early detection and providing psychological interventions with or without pharmacological interventions were found to be consistently cost-effective and even cost saving compared to usual care [41,58,73]. Preventive interventions for substance abuse resulted in a cost-benefit ratio of 1:26 due to the reduction in employee injury [59]. An indicated CBT programme for panic disorders might be a cost-effective intervention with the reported ICER of €6,198 (or $9,766) per panic disorder-free survival gained [61]. Compare to either traditional CBT or status quo for prevention of generalised anxiety disorder, mobile CBT delivered to those with mild anxiety disorder was found to be cost saving over a lifetime.

Several economic evaluations have been conducted to promote mental health and well-being on targeted populations. The majority of studies strongly supported the value for money of these interventions. Preventive interventions targeting employees (in general) or nurses with elevated risk of mental health problems were found to be cost saving, with a return of $1.5 to $7 per $1 invested, respectively [40,50,74]. A modelled implementation of an exercise referral scheme for mental health promotion in the UK over 1 year was cost-effective with an ICER of £12,111 (or $20,665) per QALY gained—well below the NICE threshold of £20,000 per QALY gained [51]. Furthermore, universal mental health promotion programmes in community settings in the UK were found to be cost saving under the societal perspective and more effective and more costly under the health sector perspective, with an ICER of £91 (or $124) per unit improvement on the depression and anxiety symptom scale. Ride and colleagues highlighted that a psychoeducational intervention targeting first-time mothers to promote mental health and well-being had an ICER of A$36,451 (or $27,679) per QALY gained [52].

#### Older adults (>65 years)

Table 3 presents the characteristics and main findings of studies focused on older adults. Seven studies assessed the cost-effectiveness of mental health prevention and promotion interventions in older adults [75–81]. Studies were conducted in the following countries: UK (*n* = 3), the Netherlands (*n* = 3), and US (*n* = 1). Three studies conducted multiple evaluation frameworks (CEA and CUA), 2 conducted a CEA, and 2 a CUA. Of those, 4 studies focused on interventions targeting depression and anxiety, 2 studies on depression only, and 1 study that assessed interventions that aimed to improve older adults’ general mental health and well-being. All 7 studies were conducted alongside randomised controlled trials, which had a follow-up period of 6 to 12 months. Three studies adopted a societal perspective, 3 studies a narrower health and social care perspective (with 1 study including informal care costs), and 1 study did not state the perspective.

**Table 3:** Characteristics of studies included—older adults.

Lead author (year), country	Targeted mental risk factor or condition/disorder	Population description (universal, selective, indicated)	Intervention(s) and comparator (setting, individual or group-based, parenting)	Evaluation type	Study design (trial (*n* =) or model)	Perspective, time horizon	Year of pricing, discount rates	Cost categories	Outcomes	Results ICERs (in 2020 US$)^a^	Limitations	Quality score^b^
Bosmans and colleagues (2014) [75], the Netherlands	Depression and anxiety	Older people living in elderly homes (targeted)	Stepped care programme Usual care	CEA CUA	RCT (*n* = 185)	Societal, 10 months	2008, not applicable	Intervention cost, healthcare costs	Incidence and severity of depression and anxiety; QALYs	ICER = €26,890 ($38,326) per QALY gained ICER = €85,521 ($121,892) per depression or anxiety case avoided ICER = −€10,293 (−$14,670) per depression case avoided ICER = €10,328 ($14,720) per anxiety case avoided ICER = €364 ($519) per improvement on CES-D ICER = €963 ($1,373) per improvement on HADS-A	Large dropout rate, especially in the intervention arm, indicates low compliance and implementation difficulty	100
Van’t Veer-Tazelaar and colleagues (2010) [81], the Netherlands	Depression and anxiety	Older people at high risk of depression and anxiety (targeted)	Stepped care preventive intervention Routine primary care	CEA	RCT (*n* = 170)	Societal, 12 months	2007, not applicable	Direct and non-direct medical costs	Disorder-free year	ICER = € 4,367 ($6,368) per disorder-free year gained	Higher dropout rate in the intervention arm	100
Joling and colleagues (2013) [77], the Netherlands	Depression and anxiety	Caregivers of people with dementia (targeted)	Family meetings intervention Usual care	CEA CUA	RCT (*n* = 192)	Societal, 12 months	2009, not applicable	Intervention cost, healthcare costs, home care costs, productivity, informal care	Incidence in anxiety and depression; QALYs	ICER = €157,534 ($224,033) per QALY dyad ICER = −€32,254 (−$45,869) per caregiver QALY ICER = €2,574,938 ($3,661,883) per patient QALY ICER = −€59011 (−$83,921) per incidence of depression and/or anxiety	Incomplete data of costs and outcomes (47% of carer data missing)	100
Knapp and colleagues (2013) [78], UK	Depression and anxiety	Family carers of people with dementia (targeted)	Eight session, manual based, coping intervention Usual care	CEA CUA	RCT (*n* = 260)	Health and social care, 8 months	2009–2010, not applicable	Intervention costs (outpatient, community, other); usual treatment (outpatient, community, other)	HADS-T; QALYs	ICER with QALY as outcome = £6000 ($10,395); ICER with HADS-T as outcome = £118 ($204)	Perspective limited to health and social care costs and only carer outcomes were considered	94
Romeo and colleagues (2011) [79], UK	Depression	Older people who have had hip fracture surgery with and without depression (targeted)	CBT + Treatment as usual vs. Treatment as usual	CEA	RCT (*n* = 170)	Health, social care, voluntary sector agencies and unpaid carers, 6 months	2005/2006, not applicable	Intervention cost, service cost, non-service cost	HADS depression score	ICER = £1,800 ($3,440) per unit improvement in HADS depression	Limited perspective, short time horizon, outcomes not in QALYs	100
Underwood and colleagues (2013) [80], UK	Depression	Care home residents with depression (targeted)	Physical intervention (whole-home intervention) Control home	CUA	RCT (*n* = 798)	12 months, National Health Service provider and societal	2010, not applicable	Community visits, GP home visit, GP surgery visit, inpatient services, medications, mental health, Outpatient services, practice nurse, accident and emergency	QALYs	ICER was not calculated as the intervention was more expensive with a net reduction in QALYs (incremental costs = £374/$639 (NHS) or £366/$625 (societal); incremental QALYs = −0.0014)	Difficulty to obtain EQ-5D data in an elderly and frail population	98
Clark and colleagues (2012) [76], US	Mental well-being	Older adults aged 60–95 years (universal)	Occupational therapy intervention (Well Elderly Lifestyle Redesign intervention) Usual care	CUA	RCT (*n* = 460)	Not stated, 6 months	Not stated (assumed 2010), not stated	Intervention cost	QALYs	ICER = US$41,218 ($49,186)	Only intervention costs were considered	45

^a^CCEMG-EPPI-Centre Cost Converter: web-based tool for adjusting estimates of cost on November 2, 2020 using IMF source dataset for PPP.

^b^Quality assessment was undertaken using the QHES checklist.

CBT, cognitive behaviour therapy; CEA, cost-effectiveness analysis; CES-D, Center for Epidemiological Studies Depression Scale; CUA, cost-utility analysis; EQ-5D, EuroQol 5-Dimension; GP, general practitioner; HADS, Hospital Anxiety and Depression Scale; ICER, incremental cost-effectiveness ratio; NHS, National Health Service; PPP, purchasing power parity; QALY, quality-adjusted life year; QHES, Quality of Health Economic Studies Instrument; RCT, randomised controlled trial.

Two studies assessed the cost-effectiveness of a stepped-care programme to prevent depression and anxiety in older adults. In the first study, the intervention was structured in cycles of 3 months and consisted of 4 steps: watchful waiting, bibliotherapy, problem-solving treatment, and antidepressant medication [81]. The authors found that the intervention reduced the incidence of depression and anxiety and—assuming a willingness to pay for a disorder-free year of €5,000—the intervention represented good value for money compared with routine primary care (€4,367 [$6,368]). Contrary to this, the second study found that a stepped care programme, consisting of watchful waiting, activity scheduling, life review, and consultations with the general practitioner, was not cost-effective in residents of homes for elderly people compared with usual care across all outcome measures (QALYs, incidence, and severity of depression and anxiety) [75].

Two studies, targeting carers of people living with dementia, examined the cost-effectiveness of a family meeting intervention [77] and an 8-session coping intervention [78] applying both a CEA and CUA framework. Compared with usual care, the family intervention consisting of 6 in-person counselling sessions was not considered cost-effective in terms of QALY gains and incidence of depression and/or anxiety in caregivers [77]. The adapted version of the “Coping with Caring” intervention, however, was cost-effective compared with treatment as usual by reference to both carer-based QALYs (£6,000 or $10,395) and affective symptoms of family carers (£118 or $204) [78].

The remaining 3 studies examined the cost-effectiveness of an universal occupational therapy intervention in older adults aged 60 to 95 years [76], a whole-home intervention that comprised training for care home staff and twice weekly physiotherapist-led exercise classes in care home residents [80], and CBT in older people who have had hip surgery [79]. Using the UK’s cost per QALY threshold (£20,000 to £30,000 per QALY gained) as reference, the authors concluded that the occupational therapy intervention was cost-effective in improving older adults’ mental well-being, whereas the whole-home intervention and CBT for prevention of depression were found not cost-effective.

### Finding synthesis

**Fig 2:**
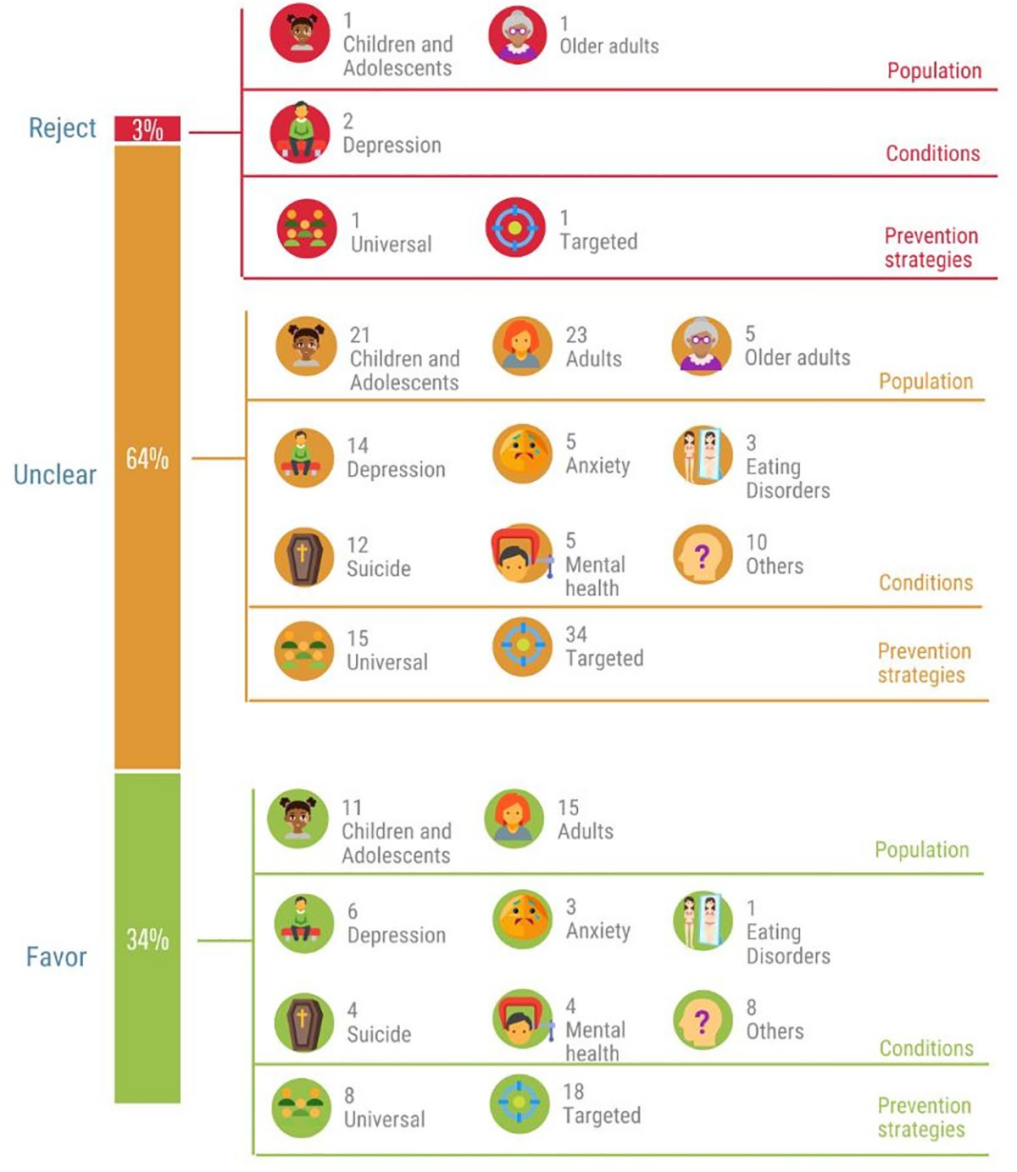
Cost-effectiveness results and implications for decision-makers.

As presented in Tables 1–3, the format and extent of reported economic evaluation frameworks, targeted population and conditions, health outcomes, and costs varied considerably between studies, precluding the aggregation of quantitative data such as meta-analysis. Therefore, the dominance ranking framework was used for qualitative synthesis of included studies (see S3 Table). Fig 2 presents a summary of the classification of different interventions graded based on costs and health benefits and grouped as either an intervention to be rejected, favoured, or unclear.

Only 2 interventions were categorised as “reject” (i.e., less effective and more costly), which were preventive interventions for depression. Particularly, one intervention was a universal school-based CBT for adolescents, while another was targeted physical intervention for older adults. One-third of interventions was ranked under interventions to be “favoured” as they yielded positive health benefits at a lower cost. These interventions focused on children, adolescents, or adults, and most of them were targeted for the prevention of depression, suicide, or promotion of mental health. The remaining interventions, accounting for nearly two-thirds of interventions, were in the “unclear” category since they produced improved health outcomes at a higher cost. Interventions classified in this unclear group required value judgements in terms of the willingness to pay threshold that often varies by decision context. Based on authors’ conclusions, over half of these interventions were considered to be cost-effective given that the ICER remained under relevant value for money threshold of $50,000 per QALY or £20,000 to £30,000 per QALY.

### Quality assessment

The quality scores ranged from 22 to 100. Only 1 study was classified as extremely poor quality [44], and 4 studies were classified as poor quality [50,56,69,76]. The majority of studies achieved fair to high quality. The average quality scores for studies focused on children, adults, and older adults were 88.2, 79.7, and 91.0 respectively. Detail of quality scores for each study is presented in S2 Table.

## Discussion

### Summary of the main findings

This review provides an update on economic evaluation studies of mental health promotion and prevention interventions across the life span. Findings from the current review highlight that there has been less research relating to the cost-effectiveness of mental health promotion interventions compared with preventative interventions. Furthermore, there is limited evidence available for low- and middle-income countries, given that the majority of studies were conducted in high-income countries, mostly in the UK, the US, or Australia. Given that childhood years are significant for building life skills and marks the time period when mental health disorders develop, it is not surprising that most of the existing research has focused on children, adolescents, and youth. There was less evidence on interventions targeting older adults. CUA or CEA were the most frequent types of economic evaluation across the age spectrum. Interestingly, although there was high heterogeneity in terms of the design among included economic evaluations, the majority of the studies consistently found that interventions for mental health prevention and promotion were cost-effective or cost saving. The review found that targeted prevention was likely to be cost-effective compared to universal prevention. In children and adolescents, screening plus psychological interventions (e.g., CBT) at school were the most cost-effective interventions for prevention of mental disorders, while parenting interventions had good evidence in mental health promotion. In adults, strong evidence supported screening plus psychological interventions for mental disorder prevention, while workplace interventions targeting employees in general were cost-effective. There is inconclusive evidence for preventive interventions for mental disorders or mental health promotion in older adults.

This review found significant growth in the number of economic evaluations for mental health prevention and promotion published in the last 10 years. The number of studies included in this review was 4 to 5 times greater compared to the numbers in the previous reviews [11,12]. It is noteworthy that the current review has a broader scope where prevention of substance use disorders and suicide have been considered. Furthermore, this review also included ROI studies, with evidence suggesting that preventive interventions for suicide and externalising problems in children/adolescents as well as for depression and substance use in adults produce significant returns.

### Methodological limitations of included studies

While there have been considerable improvements in both quantity and quality of cost-effectiveness studies in comparison to the previous reviews, there are still some persistent methodological limitations in the current studies reviewed. Some studies, especially CBA/ROI studies, did not state the perspective adopted in the study, which is critical for identification of cost components. Also, the comparator to which the intervention of interest was compared to was not clearly stated or described. Most CBA/ROI studies relied on survey data rather than controlled trials, introducing bias for judging the effectiveness and cost-effectiveness of the respective interventions.

One notable methodological issue relates to the absence of incorporating statistical uncertainty in both trial-based and modelled economic evaluations. Ideally, both deterministic and probabilistic uncertainty analyses should be conducted within a single economic evaluation to reflect the parameter uncertainty [82]. The deterministic analysis is used for reporting the impact of key parameters on the ICER, whereas probabilistic analysis is used to convey overall uncertainty. Regardless of the type of statistical uncertainty, these analyses are helpful for the analysts to examine the reliability or robustness of cost-effectiveness inferences and helps to inform the direction of further research [82,83].

Another issue pertinent to trial-based economic evaluations relates to the fact that the majority of results of effectiveness and costs did not reach statistical significance. This is most likely due to an insufficient sample size to detect statistical significance in costs or QALYs, as power calculations are often based on clinical outcomes. Another important issue for the prevention field is that a longer time horizon is required to detect the effectiveness of the intervention. However, most of the studies included were limited to a time horizon of up to 1 year, and only 2 studies had time horizons of 4 years and 15 years [36,41].

### Policy implications and directions for future research

While this review identified a number of interventions for the prevention of mental disorders and mental health promotion that provide value for money, the scale-up of such interventions requires further consideration. Firstly, most of the studies were conducted alongside a clinical trial, where efficacy estimates often do not translate into real-life effectiveness. For example, an early CEA of the early intervention programme designed to prevent anxiety and depression from preschool age (the Cool Little Kids programme) showed that the intervention is cost-effective when modelling trial efficacy results [22]. The translational trial of Cool Little Kids, which aimed to provide real-world effectiveness and cost-effectiveness evidence, resulted in higher incremental costs [84]. The studies also differed in their time horizons (3 years versus 1 year) and the choice of outcomes (DALYs versus internalising problems avoided), indicating that trial cost-effectiveness outcomes need to be interpreted carefully. The large-scale implementation of mental health promotion and prevention interventions also requires consideration beyond cost-effectiveness outcomes. For example, numerous trial-based economic evaluations reported high dropout rates, indicating problems with acceptability, adherence, and feasibility of the interventions evaluated. These implementation considerations need to be considered alongside methodological limitations of cost-effectiveness studies. There are also potential barriers that arise to implement such programmes at the policy level [85]. In particular, for interventions that impact multiple sectors, fragmentation of responsibility and funding across stakeholders and sectors may be problematic. Limited capacity to deliver such services or limited incentives to invest in prevention requires an even stronger evidence base to promote the value of investing in mental health promotion and prevention interventions.

The small number of mental health promotion studies identified as part of this review may, to some degree, be an artefact of the difficulty in conducting economic evaluations in the areas of health promotion and public health as previously highlighted [11]. In fact, given that interventions for mental health promotion are often reliant on population and public health strategies, natural experiments rather than randomised controlled trials are frequently used as a research design to evaluate such interventions if the requisite data are available [86]. Furthermore, preventive interventions or public health interventions require financial support and implementation from sectors outside of health (e.g., school-based interventions typically involve the education sector) [87]. Standard economic evaluation methods commonly applied to health technology assessment may not be transferable to health promotion evaluation and broader frameworks, such as cost-benefit analyses may be required where broader benefits can be captured and measured [88]. Given the rapid growth of the number of interventions for promoting mental or psychological well-being, and building resilience [89], economic evaluations with improved methods and capturing intersectoral cost and outcomes of such interventions are needed [86].

Further economic evaluation studies are also warranted for the promotion of good mental health and the prevention of ill-health in older adults. Only 7 studies were identified in this review, of which 2 studies focused on family carers of people living with dementia [90]. Depression among older people is especially a significant public health issue, with estimates showing that 8.2% of community-dwelling older adults [91] and 52% of older adults residing in residential aged care in Australia are experiencing depressive symptoms [92].

### Strength and limitations

To the best of our knowledge, this is the first comprehensive review that covers both prevention of mental disorders and mental health promotion across the age spectrum. This review also included ROI studies, which are commonly used by decision-makers.

A limitation of our review is that we may have missed studies that promote mental health or prevent mental disorders as part of general health promoting strategies. However, since we focused on studies where mental health was the primary outcome, studies that did not distinguish between mental and physical health could have been excluded. Examples for this scenario include numerous workplace health promotion programmes that do not address mental health directly but still may have a positive impact on mental health. Furthermore, although ROI studies were included in the search strategy, it is common that these studies are published in grey literature rather than in the academic literature. For example, several reports published by governmental agencies in the UK, Canada, and Australia were not included in this review [93,94]. However, these reports consistently supported the value for money of interventions designed for mental health prevention and promotion.

The quality assessment checklist used in this review (the QHES) may have limitations in capturing the quality of trial-based economic evaluations, given that the assessment criteria have a strong focus on the key aspects of modelled evaluations in particular. Furthermore, the inclusion of 2 or 3 questions in one assessment criterion resulted in difficulties assigning an appropriate score, especially given the lack of an option to assign a middle score for each criterion in the QHES. Studies that partly met a criterion did not achieve a score for that reason. Further research on quality assessment checklists of economic evaluations is required.

## Conclusions

Our findings suggested a significant growth in the number and quality of economic evaluations in the prevention of mental disorders or promotion of mental health and well-being. Although several interventions for mental health prevention and promotion provide good value for money, the varied quality as well as methodologies used in economic evaluations limit the generalisability of conclusions about cost-effectiveness. Further translational research of real world implementation of mental health prevention and promotion is required.

## Supporting information

S1 PRISMA Checklist Completed PRISMA checklist. (DOC)

S1 Table Search concepts and the corresponding key words used. (DOCX)

S2 Table Quality assessment results of included studies. (DOCX)

S3 Table Synthesis of findings using the dominance ranking framework. (DOCX)

## Abbreviations

CBA: cost-benefit analysis
CBT: cognitive behavioural therapy
CEA: cost-effectiveness analysis
CMA: cost-minimisation analysis
CUA: cost-utility analysis
DALY: disability-adjusted life year
ED: eating disorder
ICER: incremental cost-effectiveness ratio
MDD: major depressive disorder
NICE: National Institute for Health and Clinical Excellence
PRISMA: Preferred Reporting Items for Systematic Reviews and Meta-Analyses
QALY: quality-adjusted life year
QHES: Quality of Health Economic Studies Instrument
ROI: return on investment
