# Supplementary material for: Cost-effectiveness evidence of mental health prevention and promotion interventions: A systematic review of economic evaluations
Source: PLoS Med. 2021 May 11;18(5):e1003606. doi: 10.1371/journal.pmed.1003606 (PMC8148329; doi:10.1371/journal.pmed.1003606)
Supplement: S1 PRISMA Checklist — (DOC) [file pmed.1003606.s001.doc]

| **Section/topic** | **#** | **Checklist item** | **Reported on page #** |
| --- | --- | --- | --- |
| **TITLE** | | |  |
| Title | 1 | Identify the report as a systematic review, meta-analysis, or both. | Title, 1 |
|  |  | Cost effectiveness evidence of mental health prevention and promotion: A systematic review of economic evaluations. |  |
| **ABSTRACT** | | |  |
| Structured summary | 2 | Provide a structured summary including, as applicable: background; objectives; data sources; study eligibility criteria, participants, and interventions; study appraisal and synthesis methods; results; limitations; conclusions and implications of key findings; systematic review registration number. | Abstract, paragraph 1 |
|  |  | Background:  The prevention of mental disorders and promotion of mental health and wellbeing are growing fields. Whether mental health promotion and prevention interventions provide value for money in children, adolescents, adults and older adults is unclear. The aim of the current study is to update two existing reviews of cost-effectiveness studies in this field in order to determine whether such interventions are cost-effective.  Methods and findings:  Electronic databases (including MEDLINE, PsycINFO, CINAHL and EconLIT through EBSCO and Embase) were searched for published cost-effectiveness studies of prevention of mental disorders and promotion of mental health and wellbeing from 2008 to 2020. The quality of studies was assessed using the Quality of Health Economic Studies Instrument. The protocol was registered with PROSPERO (# CRD42019127778). The primary outcomes were incremental cost effectiveness ratio or return on investment ratio across all studies.  Sixty-five studies met the inclusion criteria of a full economic evaluation, of which, 23 targeted children and adolescents, 35 targeted adults while the remaining targeted older adults. A large number of studies focused on prevention of depression and/or anxiety disorders, followed by promotion of mental health and well-being and other mental disorders. Although there was high heterogeneity in terms of the design among included economic evaluations, most studies consistently found that interventions for mental health prevention and promotion were cost-effective or cost saving. The review found that targeted prevention was likely to be cost effective compared to universal prevention. Screening plus psychological interventions (e.g. Cognitive Behavioural Therapy) at school were the most cost-effective interventions for prevention of mental disorders in children and adolescents while parenting interventions and workplace interventions had good evidence in mental health promotion. There is inconclusive evidence for preventive interventions for mental disorders or mental health promotion in older adults. While studies were of general high quality, there was limited evidence available from low and middle-income countries.  The review was limited to studies where mental health was the primary outcome and may have missed general health promoting strategies that could also prevent mental disorder or promote mental health. Some return-on-investment studies might not be included given that these studies are commonly published in grey literature rather than in the academic literature.  Conclusions:  Our review found a significant growth of economic evaluations in prevention of mental disorders or promotion of mental health and well-being over the last 10 years. Although several interventions for mental health prevention and promotion provide good value-for-money, the varied quality as well as methodologies used in economic evaluations limit the generalisability of conclusions about cost-effectiveness. However, the finding that the majority of studies especially in children, adolescents and adults demonstrated good value for money is promising. . Research on cost effectiveness in low-middle income settings is required. |  |
| **INTRODUCTION** | | |  |
| Rationale | 3 | Describe the rationale for the review in the context of what is already known. | Introduction, Paragraph 3 |
|  |  | There have been two published reviews of economic evaluations of preventive interventions, focusing on the prevention of mental disorders and the promotion of mental health. Zechmeister et al. 2008 found seven studies targeting children and adolescents in a wide range of interventions, such as cognitive behavioral therapy (CBT), peer or crisis support, social work intervention and early child development programs that have strong evidence of cost-effectiveness [11]. Mihalopoulos and Chatterton 2015 updated that review and found that even though the evidence on the cost effectiveness of mental health intervention was markedly increasing, there was often a lack of comparability across studies [12]. An important limitation of the review by Mihalopoulos and Chatterton 2015 was the focus on preventive interventions for mental disorders and did not include mental health promotion types of interventions as well as ROI studies [12]. Given that there is a growing interest in these types of studies for decision-makers and more economic evaluations have been published since the last review, the aim of this study is to provide an update of the current literature on the cost-effectiveness of mental health promotion and prevention interventions across the age spectrum. The current review will answer a critical question whether health promotion and prevention provide value for money compared to no intervention in children, adolescents, adults and older adults. |  |
| Objectives | 4 | Provide an explicit statement of questions being addressed with reference to participants, interventions, comparisons, outcomes, and study design (PICOS). | Introduction, Paragraph 3 |
|  |  | Given that there is a growing interest in these types of studies for decision-makers and more economic evaluations have been published since the last review, the aim of this study is to provide an update of the current literature on the cost-effectiveness of mental health promotion and prevention interventions across the age spectrum. The current review will answer a critical question whether health promotion and prevention provide value for money compared to no intervention in children, adolescents, adults and older adults. |  |
| **METHODS** | | |  |
| Protocol and registration | 5 | Indicate if a review protocol exists, if and where it can be accessed (e.g., Web address), and, if available, provide registration information including registration number. | Methods, paragraph 1 |
|  |  | This systematic review protocol was registered on the PROSPERO database (registration number: CRD42019127778). |  |
| Eligibility criteria | 6 | Specify study characteristics (e.g., PICOS, length of follow-up) and report characteristics (e.g., years considered, language, publication status) used as criteria for eligibility, giving rationale. | Methods, Identification and selection of studies, paragraph 2 |
|  |  | Studies were included if they undertook an economic evaluation or a ROI study (i.e., at least two interventions examining both costs and benefits). This excluded partial economic evaluations (i.e., studies that had no comparator or studies that only focused on costs or benefits but not on both). Furthermore, studies related to treatment rather than prevention or promotion were also excluded. The review also focused on studies that only reported mental health conditions (e.g. anxiety disorder), symptoms (e.g. anxiety level) or risk factors (e.g. bullying) as the primary outcome. Studies published before 2008, not in peer-reviewed journal articles, and in languages other than English were excluded. Included studies were categorized according to children and adolescents (aged 0 to 18), adults (aged 18 to 65) and older adults (aged 65 and above). If a study included a mixed population, it was classified based on the mean age of the population included in the study. Studies were categorized into ’prevention; that assessed the cost-effectiveness of an intervention that aimed to reduce the incidence, prevalence or seriousness of mental health problems and illness while mental health ‘promotion’ comprised studies that examined the cost-effectiveness of interventions that focused on increasing mental well-being, enhancing positive mental health and empowering individuals and communities. |  |
| Information sources | 7 | Describe all information sources (e.g., databases with dates of coverage, contact with study authors to identify additional studies) in the search and date last searched. | Methods, Identification and selection of studies, paragraph 1 |
|  |  | An extensive literature search was conducted using electronic databases that included MEDLINE, PsycINFO, CINAHL and EconLIT through EBSCO and Embase from January 2008 to October 04, 2020. The search terms used in all these searches were organized into three blocks including (i) mental health disorders (e.g. depression, anxiety, eating disorders) and risk factors (e.g. sleep, resilience, bullying), (ii) promotion and prevention (prevention or promotion), and (iii) economic evaluation (e.g. cost-effectiveness, cost utility analysis). Further details of search terms can be obtained from Appendix - Table S1 |  |
| Search | 8 | Present full electronic search strategy for at least one database, including any limits used, such that it could be repeated. | Supporting information S1 |
|  |  | An extensive literature search was conducted using electronic databases that included MEDLINE, PsycINFO, CINAHL and EconLIT through EBSCO and Embase from January 2008 to October 04, 2020. The search terms used in all these searches were organized into three blocks including (i) mental health disorders (e.g. depression, anxiety, eating disorders) and risk factors (e.g. sleep, resilience, bullying), (ii) promotion and prevention (prevention or promotion), and (iii) economic evaluation (e.g. cost-effectiveness, cost utility analysis). Further details of search terms can be obtained from Appendix - Table S1  **Table S1. Search concepts and the corresponding key words used** |  |
| Study selection | 9 | State the process for selecting studies (i.e., screening, eligibility, included in systematic review, and, if applicable, included in the meta-analysis). | Methods, Identification and selection of studies, paragraph 1 & 2 |
|  |  | An extensive literature search was conducted using electronic databases that included MEDLINE, PsycINFO, CINAHL and EconLIT through EBSCO and Embase from January 2008 to October 04, 2020. The search terms used in all these searches were organized into three blocks including (i) mental health disorders (e.g. depression, anxiety, eating disorders) and risk factors (e.g. sleep, resilience, bullying), (ii) promotion and prevention (prevention or promotion), and (iii) economic evaluation (e.g. cost-effectiveness, cost utility analysis). Further details of search terms can be obtained from Appendix - Table S1. All citations were imported into an electronic database (Endnote version X8 [13]) in which the duplications were eliminated. A screening web-tool system, RAYYAN [14] , was then used for the screening process. The retrieved studies were split into two groups; each group of references was screened by two reviewers (i.e., group one screened by ACE and LE; group two screened by ACE and LL). A third reviewer from the other allocated group resolved any variation in decisions.  Studies were included if they undertook an economic evaluation or a ROI study (i.e., at least two interventions examining both costs and benefits). This excluded partial economic evaluations (i.e., studies that had no comparator or studies that only focused on costs or benefits but not on both). Furthermore, studies related to treatment rather than prevention or promotion were also excluded. The review also focused on studies that only reported mental health conditions (e.g. anxiety disorder), symptoms (e.g. anxiety level) or risk factors (e.g. bullying) as the primary outcome. Studies published before 2008, not in peer-reviewed journal articles, and in languages other than English were excluded. Included studies were categorized according to children and adolescents (aged 0 to 18), adults (aged 18 to 65) and older adults (aged 65 and above). If a study included a mixed population, it was classified based on the mean age of the population included in the study. Studies were categorized into ’prevention; that assessed the cost-effectiveness of an intervention that aimed to reduce the incidence, prevalence or seriousness of mental health problems and illness while mental health ‘promotion’ comprised studies that examined the cost-effectiveness of interventions that focused on increasing mental well-being, enhancing positive mental health and empowering individuals and communities. |  |
| Data collection process | 10 | Describe method of data extraction from reports (e.g., piloted forms, independently, in duplicate) and any processes for obtaining and confirming data from investigators. | Methods, Data extraction, Paragraph 1 |
|  |  | Characteristics of the studies were extracted into a standardized table that was adapted from previous reviews of economic evaluations and the review guideline for economic evaluations developed by the Joanna Briggs Institute [12, 15-17]. The data extraction table included: characteristics of the population, country, perspective, type of prevention (universal, selective and indicated prevention), time-horizon, type of economic evaluation (i.e. CUA, CEA, CBA or ROI), study design (i.e. modelled or randomized controlled trial), outcome measures (e.g. QALYs, life years saved, incidence or severity of clinical outcomes) and cost-effectiveness results (the incremental cost effectiveness ratio - ICER or ROI ratio). To make a relevant comparison of the ICERs across the identified studies, all costs were converted into 2020 US dollars. The CCEMG – EPPI-Centre Cost Converter version 1.4 that uses the purchasing power parity approach sourced from the IMF World Economic Outlook Database was used to convert all non-US dollar currencies to US Dollar currencies [18]. For studies that did not report the reference year, an assumption of two years prior to the publication date was made as the base year. Data extraction was undertaken by ACE and OC and double-checked by LL and LE. Disagreements were resolved by discussion between two review authors (LL and LE). |  |
| Data items | 11 | List and define all variables for which data were sought (e.g., PICOS, funding sources) and any assumptions and simplifications made. | Methods, Data extraction, paragraph 1 |
|  |  | Characteristics of the studies were extracted into a standardized table that was adapted from previous reviews of economic evaluations and the review guideline for economic evaluations developed by the Joanna Briggs Institute [12, 15-17]. The data extraction table included: characteristics of the population, country, perspective, type of prevention (universal, selective and indicated prevention), time-horizon, type of economic evaluation (i.e. CUA, CEA, CBA or ROI), study design (i.e. modelled or randomized controlled trial), outcome measures (e.g. QALYs, life years saved, incidence or severity of clinical outcomes) and cost-effectiveness results (the incremental cost effectiveness ratio - ICER or ROI ratio). To make a relevant comparison of the ICERs across the identified studies, all costs were converted into 2020 US dollars. The CCEMG – EPPI-Centre Cost Converter version 1.4 that uses the purchasing power parity approach sourced from the IMF World Economic Outlook Database was used to convert all non-US dollar currencies to US Dollar currencies [18]. For studies that did not report the reference year, an assumption of two years prior to the publication date was made as the base year. Data extraction was undertaken by ACE and OC and double-checked by LL and LE. Disagreements were resolved by discussion between two review authors (LL and LE). |  |
| Risk of bias in individual studies | 12 | Describe methods used for assessing risk of bias of individual studies (including specification of whether this was done at the study or outcome level), and how this information is to be used in any data synthesis. | Methods – Quality assessment, paragraph 1 |
|  |  | Quality assessment  The Quality of Health Economic Studies Instrument (QHES) was used to assess the quality of included studies [19]. The checklist consists of 16 questions, to be answered with yes or no, and each question is weighted based on importance. Given the lack of a ‘not applicable’ option in the original QHES, we decided that if a question from the QHES was not applicable for a particular study (e.g. the study was a trial-based economic evaluation while the question was related to modelled evaluations), this question was answered with ‘yes’. Regarding the inclusion of two or three questions in one-assessment criterion in the QHES, studies that partly met a criterion did not achieve a score. The quality score was calculated by adding up all of the points for the questions answered “Yes”. Cut-off points were used to determine the quality: 0-24 (extremely poor quality); 25-49 (poor quality); 50-74 (fair quality); 75-100 (high quality). Quality assessment was undertaken by ACE and OC and double-checked by LL and LE. Disagreements were resolved by discussion between two review authors (LL and LE). |  |
| Summary measures | 13 | State the principal summary measures (e.g., risk ratio, difference in means). | Methods, Data synthesis, paragraph 1 |
|  |  | Data synthesis  Economic findings were synthesized and presented as a narrative summary in conjunction with a tabular summary. Given that there is high heterogeneity in terms of population, intervention, comparator and outcome as well as economic evaluation frameworks across included studies, a meta-analysis was not conducted. Instead, the dominance ranking framework (or permutation matrix) presenting the distribution of studies across nine possible outcomes in terms of costs and effectiveness was adapted from the systematic review of economic evaluation guidelines developed by the Joanna Briggs Institute [17]. In the dominance raking framework, colour coding was used to indicate implications for decision makers. A ’red’ coding shows the situation in which a decision is less favoured or rejected by decision makers (i.e. costs are higher and the intervention is less effective). A ‘green’ code indicates the case in which the intervention is strongly favoured (i.e. has better health outcomes and lower costs). A ‘yellow’ coding shows that there is no obvious decision that the intervention is more effective and more costly or less effective and less costly). That is, some form of financial or clinical trade-off is required or a value for money threshold to determine whether the intervention is cost effective. In cases where an economic evaluation evaluates two or more interventions compared to a control, results for each intervention versus no intervention or waitlist control was reported separately in the dominance framework table. Similarly, if the study reported results by different perspectives or for different outcome measures, results were reported separately and were ranked ‘unclear’ if the results were conflicting. |  |
| Synthesis of results | 14 | Describe the methods of handling data and combining results of studies, if done, including measures of consistency (e.g., I^2^) for each meta-analysis. | Methods – data synthesis, paragraph 1 |
|  |  | Data synthesis  Economic findings were synthesized and presented as a narrative summary in conjunction with a tabular summary. Given that there is high heterogeneity in terms of population, intervention, comparator and outcome as well as economic evaluation frameworks across included studies, a meta-analysis was not conducted. Instead, the dominance ranking framework (or permutation matrix) presenting the distribution of studies across nine possible outcomes in terms of costs and effectiveness was adapted from the systematic review of economic evaluation guidelines developed by the Joanna Briggs Institute [17]. In the dominance raking framework, colour coding was used to indicate implications for decision makers. A ’red’ coding shows the situation in which a decision is less favoured or rejected by decision makers (i.e. costs are higher and the intervention is less effective). A ‘green’ code indicates the case in which the intervention is strongly favoured (i.e. has better health outcomes and lower costs). A ‘yellow’ coding shows that there is no obvious decision that the intervention is more effective and more costly or less effective and less costly). That is, some form of financial or clinical trade-off is required or a value for money threshold to determine whether the intervention is cost effective. In cases where an economic evaluation evaluates two or more interventions compared to a control, results for each intervention versus no intervention or waitlist control was reported separately in the dominance framework table. Similarly, if the study reported results by different perspectives or for different outcome measures, results were reported separately and were ranked ‘unclear’ if the results were conflicting. |  |

Page 1 of 2

| **Section/topic** | **#** | **Checklist item** | **Reported on page #** |
| --- | --- | --- | --- |
| Risk of bias across studies | 15 | Specify any assessment of risk of bias that may affect the cumulative evidence (e.g., publication bias, selective reporting within studies). | Methods – quality assessment Paragraph 1 |
|  |  | Quality assessment  The Quality of Health Economic Studies Instrument (QHES) was used to assess the quality of included studies [19]. The checklist consists of 16 questions, to be answered with yes or no, and each question is weighted based on importance. Given the lack of a ‘not applicable’ option in the original QHES, we decided that if a question from the QHES was not applicable for a particular study (e.g. the study was a trial-based economic evaluation while the question was related to modelled evaluations), this question was answered with ‘yes’. Regarding the inclusion of two or three questions in one-assessment criterion in the QHES, studies that partly met a criterion did not achieve a score. The quality score was calculated by adding up all of the points for the questions answered “Yes”. Cut-off points were used to determine the quality: 0-24 (extremely poor quality); 25-49 (poor quality); 50-74 (fair quality); 75-100 (high quality). Quality assessment was undertaken by ACE and OC and double-checked by LL and LE. Disagreements were resolved by discussion between two review authors (LL and LE). |  |
| Additional analyses | 16 | Describe methods of additional analyses (e.g., sensitivity or subgroup analyses, meta-regression), if done, indicating which were pre-specified. | N/A |
| **RESULTS** | | |  |
| Study selection | 17 | Give numbers of studies screened, assessed for eligibility, and included in the review, with reasons for exclusions at each stage, ideally with a flow diagram. | Results – paragraph 1 |
|  |  | The literature search identified 4,604 articles. After excluding duplicate studies, 2,822 studies remained for title and abstract screening. The screening based on title and abstract resulted in 138 eligible studies for full-text screening. Most studies were then excluded because they did not meet the “full economic evaluation” criterion, were not primary studies reporting results of an economic evaluation (e.g. reviews), or focused on treatment rather than prevention or promotion. After the full-text screening, sixty-five studies were included for data extraction and quality assessment. Further details are presented in the PRISMA flow diagram (Fig 1). |  |
| Study characteristics | 18 | For each study, present characteristics for which data were extracted (e.g., study size, PICOS, follow-up period) and provide the citations. | Table 1, 2 and 3 |
|  |  | Please refer to Table 1, 2 and 3. |  |
| Risk of bias within studies | 19 | Present data on risk of bias of each study and, if available, any outcome level assessment (see item 12). | Table 1, 2 and 3 with Table S2 |
|  |  | Please refer to Table 1, 2 and 3 and supplementing information Table S2 |  |
| Results of individual studies | 20 | For all outcomes considered (benefits or harms), present, for each study: (a) simple summary data for each intervention group (b) effect estimates and confidence intervals, ideally with a forest plot. | Table 1, 2 and 3 |
|  |  | Please refer to Table 1, 2 and 3. |  |
| Synthesis of results | 21 | Present results of each meta-analysis done, including confidence intervals and measures of consistency. | Results – Finding synthesis, paragraph 1 |
|  |  | Finding synthesis.  As presented in Tables 1, 2 and 3, the format and extent of reported economic evaluation frameworks, targeted population and conditions, health outcomes and costs varied considerably between studies, precluding the aggregation of quantitative data such as meta-analysis. Therefore, the dominance ranking framework was used for qualitative synthesise of included studies (see Appendix Table S3). Figure 2 presents a summary of the classification of different interventions graded based on costs and health benefits and grouped as either an intervention to be rejected, favoured or unclear.  Only two interventions were categorised as ‘reject’ (i.e. less effective and more costly), which were preventive interventions for depression. Particularly, one intervention was a universal school-based CBT for adolescents while another was targeted physical intervention for older adults. One third of interventions was ranked under interventions to be ‘favoured’ as they yielded positive health benefits at a lower cost. These interventions focused on children, adolescents or adults and most of them were targeted for the prevention of depression, suicide or promotion of mental health. The remaining interventions, accounting for nearly two thirds of interventions, were in the ‘unclear’ category since they produced improved health outcomes at a higher cost. Interventions classified in this unclear group required value judgements in terms of the willingness to pay threshold that often varies by decision context. Based on authors’ conclusions, over half of these interventions were considered to be cost effective given that the ICER remained under relevant value for money threshold of $50,000 per QALY or £20,000 to £30,000 per QALY. |  |
| Risk of bias across studies | 22 | Present results of any assessment of risk of bias across studies (see Item 15). | Results – Quality Assessment, paragraph 1 |
|  |  | Quality assessment  The quality scores ranged from 22 to 100. Only one study was classified as extremely poor quality [43] and four studies were classified as poor quality [49, 55, 68, 75]. The majority of studies achieved fair to high quality. The average quality scores for studies focused on children, adults and older adults were 88.2, 79.7 and 91.0 respectively. Detail of quality scores for each study is presented in Table S2. |  |
| Additional analysis | 23 | Give results of additional analyses, if done (e.g., sensitivity or subgroup analyses, meta-regression [see Item 16]). | N/A |
| **DISCUSSION** | | |  |
| Summary of evidence | 24 | Summarize the main findings including the strength of evidence for each main outcome; consider their relevance to key groups (e.g., healthcare providers, users, and policy makers). | Discussion – Summary of the main findings section Paragraph 1 |
|  |  | This review provides an update on economic evaluation studies of mental health promotion and prevention interventions across the life span. Findings from the current review highlight that there has been less research relating to the cost-effectiveness of mental health promotion interventions compared with preventative interventions. Furthermore, there is limited evidence available for low and middle-income countries, given that the majority of studies were conducted in high-income countries, mostly in the UK, the US, or Australia. Given that childhood years are significant for building life skills and marks the time-period when mental health disorders develop, it is not surprising that most of the existing research has focused on children, adolescents and youth. There was less evidence on interventions targeting older adults. CUA or CEA were the most frequent types of economic evaluation across the age spectrum. Interestingly, although there was high heterogeneity in terms of the design among included economic evaluations, the majority of the studies consistently found that interventions for mental health prevention and promotion were cost-effective or cost saving. The review found that targeted prevention was likely to be cost effective compared to universal prevention. In children and adolescents, screening plus psychological interventions (e.g. CBT) at school were the most cost-effective interventions for prevention of mental disorders while parenting interventions had good evidence in mental health promotion. In adults, strong evidence supported screening plus psychological interventions for mental disorder prevention while workplace interventions targeting employees in general were cost-effective. There is inconclusive evidence for preventive interventions for mental disorders or mental health promotion in older adults.  This review found significant growth in the number of economic evaluations for mental health prevention and promotion published in the last 10 years. The number of studies included in this review was four to five times greater compared to the numbers in the previous reviews [11, 12]. It is noteworthy that the current review has a broader scope where prevention of substance use disorders and suicide have been considered. Furthermore, this review also included ROI studies, with evidence suggesting that preventive interventions for suicide and externalizing problems in children/adolescents as well as for depression and substance use in adults produce significant returns |  |
| Limitations | 25 | Discuss limitations at study and outcome level (e.g., risk of bias), and at review-level (e.g., incomplete retrieval of identified research, reporting bias). | Discussion – Strength and limitations section Paragraph 1 |
|  |  | Strength and limitations  This is the first comprehensive review that covers both prevention of mental disorders and mental health promotion across the age spectrum. This review also included ROI studies, which are commonly used by decision-makers.  A limitation of our review is that we may have missed studies that promote mental health or prevent mental disorders as part of general health promoting strategies. However, since we focused on studies where mental health was the primary outcome, studies that did not distinguish between mental and physical health could have been excluded. Examples for this scenario include numerous workplace health promotion programmes that do not address mental health directly but still may have a positive impact on mental health. Furthermore, although ROI studies were included in the search strategy, it is common that these studies are published in grey literature rather than in the academic literature. For example, several reports published by governmental agencies in the UK, Canada and Australia were not included in this review [89, 90]. However, these reports consistently supported the value for money of interventions designed for mental health prevention and promotion.  The quality assessment checklist used in this review (the QHES) may have limitations in capturing the quality of trial based economic evaluations, given that the assessment criteria have a strong focus on the key aspects of modelled evaluations in particular. Furthermore, the inclusion of two or three questions in one-assessment criteria resulted in difficulties assigning an appropriate score, especially given the lack of an option to assign a middle score for each criterion in the QHES. Studies that partly met a criterion did not achieve a score for that reason. Further research on quality assessment checklists of economic evaluations is required. |  |
| Conclusions | 26 | Provide a general interpretation of the results in the context of other evidence, and implications for future research. | Conclusion section Paragraph 1 |
|  |  | CONCLUSION  Our findings suggested a significant growth in the number and quality of economic evaluations in the prevention of mental disorders or promotion of mental health and well-being. Results of this review are promising, suggesting that the prevention of mental disorders and mental health promotion are likely to provide good value-for-money. Further translational research of real world implementation of mental health prevention and promotion is required. |  |
| **FUNDING** | | |  |
| Funding | 27 | Describe sources of funding for the systematic review and other support (e.g., supply of data); role of funders for the systematic review. | Acknowledgement |
|  |  | Financial support.  The earlier version of this review was funded by the National Mental Health Commission of Australia. |  |

*From:*  Moher D, Liberati A, Tetzlaff J, Altman DG, The PRISMA Group (2009). Preferred Reporting Items for Systematic Reviews and Meta-Analyses: The PRISMA Statement. PLoS Med 6(7): e1000097. doi:10.1371/journal.pmed1000097

For more information, visit: **www.prisma-statement.org**.

Page 2 of 2
