# Supplementary material for: Cost-effectiveness evidence of mental health prevention and promotion interventions: A systematic review of economic evaluations
Source: PLoS Med. 2021 May 11;18(5):e1003606. doi: 10.1371/journal.pmed.1003606 (PMC8148329; doi:10.1371/journal.pmed.1003606)
Supplement: S1 Table — (DOCX) [file pmed.1003606.s002.docx]

**S1 Table. Search concepts and the corresponding key words used**

| Concept 1 | Mental Health Disorders and risk factors | “Mental Disorder*” OR “Mental illness*” OR “Mental health” OR Mental OR Depression OR depressive OR Anxiety OR “eating disorder*” OR Bipolar OR Schizophrenia OR “Attention Deficit Hyperactivity Disorder*” OR “Attention Deficit Disorder*” OR “oppositional defiant disorder*” OR schizoaffective OR mania OR manic OR “adjustment disorder*” OR somatoform OR sleep OR neurotic OR neurosis OR “gender identit*” OR hyperkinetic OR “attachment disorder*” OR enuresis OR encopresis OR trichotillomania OR Resilience OR “social psychology” OR Positive Psychology OR Stress OR “coping behavior*” OR “mental well*” OR “positive mental health” OR “coping mechanism” OR “coping strateg*” OR “social emotional” OR loneliness OR “social isolation” OR Behavioural Disorder OR Dissociation Disorders OR “Dissociative Disorder*” OR Psychosis OR “Obsessive Compulsive Disorder” OR Delusion* OR OR Mood OR “Personality Disorder*” OR Psychosis OR “Affective Disorder*” OR Phobi* OR “Borderline Personality Disorder*”OR “Conduct Disorder*” OR “Emotional Abuse” OR hallucination OR “Personality Disorder*” OR OR “Bulimia Nervosa” OR “Antisocial behavio*”OR “Anorexia Nervosa” OR suicide OR “Obsessive compulsive Disorder*” OR Panic OR “Post traumatic stress disorder*” OR Psychotic OR Self-harm OR Stigma OR “Drug Abuse” OR Suicidal OR suicid* OR selfharm* OR self-harm* OR (self AND harm*) OR selfinjur* OR self-injur* OR (self AND injur*) OR selfpoison* OR self-poison* OR (self AND poison*) OR selfcut* OR self-cut* or (self AND cut*) OR parasuicid* OR para-suicid* or ((deliberat* or intent*) AND overdos*) OR alcohol OR substance* OR addiction OR marijuana |
| --- | --- | --- |
| Concept 2 | Promotion and Prevention | Prevent* OR Promot* |
| Concept 3 | Health Economic terms | “Value for Money” OR “Economic evaluation*” OR “Cost Effective Analys?s OR “Cost Utility Analys?s” OR “Cost Benefit Analys?s” OR “Cost Consequence* Analys?s OR “cost minimi?ation analys?s” OR “Return o? Investment” OR “return to investment” OR “Social Return o? Investment” OR “social return to investment” OR “cost effective*” OR efficien* OR “cost saving*” OR “cost analys?s” OR “return on” |
