# Supplementary material for: Cost-effectiveness evidence of mental health prevention and promotion interventions: A systematic review of economic evaluations
Source: PLoS Med. 2021 May 11;18(5):e1003606. doi: 10.1371/journal.pmed.1003606 (PMC8148329; doi:10.1371/journal.pmed.1003606)
Supplement: S2 Table — (DOCX) [file pmed.1003606.s003.docx]

**S2 TABLE. QUALITY ASSESSMENT RESULTS OF INCLUDED STUDIES USING THE QUALITY OF HEALTH ECONOMIC STUDIES**

|  | Q1. Study objective | Q2. Perspective | Q3. Variables source | | Q4. Subgroup analysis | | Q5. Uncertainty | Q6. Incremental analysis | Q7. Data abstraction | Q8. Time horizon + discounting | Q9. Cost measurement | Q10. Primary outcome | Q11. Valid outcome measures | Q12. Transparent model structure | Q13. Model choice & assumptions | Q14. Biases | Q15. Conclusions justified | Q16. Source of funding | Score |
| --- | --- | --- | --- | --- | --- | --- | --- | --- | --- | --- | --- | --- | --- | --- | --- | --- | --- | --- | --- |
|  | **7** | **4** | **8** | | **1** | | **9** | **6** | **5** | **7** | **8** | **6** | **7** | **8** | **7** | **6** | **8** | **3** |  |
| **Children and Adolescents** | | | | | | | | | | | | | | | | | | | |
| Anderson (2014) | 7 | 4 | 8 | | 1 | | 0 | 6 | 0 | 7 | 8 | 6 | 7 | 8 (T) | 7 (T) | 6 | 8 | 3 | 86 |
| Lee (2016) | 7 | 4 | 8 | | 1 | | 9 | 6 | 5 | 7 | 8 | 6 | 7 | 8 | 7 | 6 | 8 | 3 | 100 |
| Mihalopoulous (2012) | 7 | 4 | 8 | | 1 | | 9 | 6 | 0 | 7 | 8 | 6 | 7 | 0 | 7 | 6 | 8 | 3 | 87 |
| Philippssons (2013) | 7 | 4 | 8 | | 1 | | 9 | 6 | 5 | 7 | 8 | 6 | 7 | 8 (T) | 7 (T) | 6 | 8 | 3 | 100 |
| Stallard (2013) | 7 | 4 | 8 | | 1 | | 9 | 6 | 5 | 7 | 8 | 6 | 7 | 8 (T) | 7 (T) | 6 | 8 | 3 | 100 |
| Lynch (2019) | 7 | 4 | 8 | | 1 | | 9 | 6 | 5 | 7 | 8 | 6 | 0 | 8 (T) | 7 (T) | 6 | 8 | 3 | 93 |
| Ssegonja (2020) | 7 | 4 | 8 | | 1 | | 9 | 6 | 5 | 7 | 8 | 6 | 7 | 8 | 7 | 6 | 8 | 3 | 100 |
| Mihalopoulous (2015) | 7 | 4 | 8 | | 1 | | 9 | 6 | 5 | 7 | 8 | 6 | 7 | 0 | 7 | 6 | 8 | 3 | 92 |
| Simon (2012) | 7 | 4 | 8 | | 1 | | 9 | 6 | 5 | 7 | 8 | 6 | 7 | 8 (T) | 7 (T) | 6 | 8 | 3 | 100 |
| Simon (2013) | 7 | 4 | 8 | | 1 | | 9 | 6 | 5 | 7 | 8 | 6 | 7 | 8 | 7 | 6 | 8 | 3 | 100 |
| Chatterton (2020) | 7 | 4 | 8 | | 1 | | 9 | 6 | 0 | 0 | 8 | 6 | 7 | 8 (T) | 7 (T) | 6 | 8 | 3 | 88 |
| Ahern (2018) | 7 | 4 | | 0 | | 1 | 9 | 6 | 5 | 7 | 8 | 6 | 7 | 8 (T) | 7 (T) | 6 | 8 | 3 | 92 |
| Kinchin 2020 | 7 | 4 | | 8 | | 0 | 0 | 6 | 5 | 7 | 8 | 6 | 7 | 8 | 7 | 6 | 8 | 3 | 90 |
| Godoy Garraza (2018) | 7 | 4 | | 8 | | 1 | 9 | 0 | 0 | 7 | 8 | 6 | 7 | 8 | 7 | 6 | 8 | 3 | 89 |
| Gray, 2011 | 7 | 4 | | 8 | | 0 | 0 | 0 | 5 | 7 | 8 | 6 | 7 | 8 (T) | 7 (T) | 0 | 8 | 3 | 78 |
| Le (2017) | 7 | 4 | | 8 | | 1 | 9 | 6 | 5 | 7 | 8 | 6 | 7 | 8 | 7 | 6 | 8 | 3 | 100 |
| Wang (2011) | 7 | 4 | | 0 | | 0 | 9 | 0 | 0 | 7 | 8 | 6 | 7 | 0 | 0 | 6 | 8 | 3 | 65 |
| Beckman (2015) | 7 | 4 | | 8 | | 1 | 9 | 6 | 0 | 7 | 8 | 6 | 7 | 8 | 7 | 6 | 8 | 3 | 95 |
| Deogan (2015) | 7 | 4 | | 8 | | 1 | 9 | 6 | 5 | 7 | 8 | 6 | 7 | 8 | 0 | 0 | 8 | 3 | 87 |
| Ocasio, 2014 | 7 | 0 | | 0 | | 0 | 0 | 0 | 5 | 0 | 8 | 6 | 7 | 8 (T) | 7 (T) | 6 | 8 | 3 | 65 |
| Nystrand (2019) | 7 | 4 | | 8 | | 0 | 9 | 6 | 5 | 7 | 8 | 6 | 7 | 8 | 7 | 0 | 8 | 3 | 94 |
| Dalziel, 2015 | 7 | 4 | | 8 | | 0 | 9 | 6 | 5 | 7 | 8 | 6 | 7 | 8 (T) | 7 (T) | 0 | 8 | 3 | 93 |
| Herman (2015) | 7 | 4 | | 8 | | 0 | 9 | 6 | 0 | 7 | 8 | 0 | 0 | 8 (T) | 7 (T) | 6 | 8 | 3 | 81 |
| **Adults** | | | | | | | | | | | | | | | | | | | |
| Kumar (2018) | 7 | 4 | | 8 | | 4 | 0 | 6 | 5 | 7 | 8 | 6 | 0 | 8 | 7 | 6 | 8 | 3 | 84 |
| Lintvedt (2013) | 7 | 0 | | 8 | | 1 | 0 | 0 | 5 | 0 | 0 | 0 | 0 | 0 | 0 | 6 | 8 | 0 | 35 |
| Dukhovny, (2013) | 7 | 4 | | 8 | | 1 | 9 | 6 | 5 | 7 | 8 | 6 | 7 | 8(T) | 7 (T) | 6 | 8 | 3 | 100 |
| Henderson (2019) | 7 | 4 | | 8 | | 1 | 9 | 6 | 5 | 0 | 8 | 6 | 7 | 8 (T) | 7 (T) | 6 | 8 | 3 | 93 |
| Lokkerbol (2014) | 7 | 0 | | 8 | | 0 | 0 | 0 | 5 | 7 | 0 | 0 | 7 | 8 | 7 | 6 | 8 | 3 | 66 |
| Mihalopoulos (2011) | 7 | 4 | | 8 | | 1 | 9 | 6 | 5 | 7 | 8 | 6 | 7 | 8 | 7 | 6 | 8 | 3 | 100 |
| Buntrock (2017) | 7 | 4 | | 8 | | 1 | 9 | 6 | 5 | 7 | 8 | 6 | 7 | 8 (T) | 7 (T) | 6 | 8 | 3 | 100 |
| Hunter (2014) | 7 | 4 | | 8 | | 1 | 9 | 6 | 5 | 0 | 8 | 6 | 7 | 8 | 7 | 6 | 8 | 3 | 93 |
| Van den Berg (2011) | 7 | 4 | | 8 | | 1 | 9 | 6 | 5 | 7 | 8 | 6 | 7 | 8 | 7 | 6 | 8 | 3 | 100 |
| Fernandez (2018) | 7 | 4 | | 8 | | 1 | 9 | 6 | 5 | 7 | 8 | 6 | 7 | 8 (T) | 7 (T) | 6 | 8 | 3 | 100 |
| Jiao (2017) | 7 | 4 | | 8 | | 1 | 9 | 6 | 5 | 0 | 8 | 6 | 7 | 0 | 7 | 6 | 8 | 3 | 85 |
| Goetzel (2014) | 7 | 0 | | 0 | | 1 | 0 | 6 | 5 | 7 | 0 | 6 | 7 | 8 (T) | 7 (T) | 6 | 8 | 3 | 75 |
| Ising (2015), Ising (2017) | 7 | 4 | | 8 | | 1 | 9 | 6 | 5 | 7 | 8 | 6 | 7 | 8 (T) | 7 (T) | 6 | 8 | 3 | 100 |
| Akers (2017) | 7 | 0 | | 8 | | 1 | 0 | 6 | 0 | 0 | 8 | 0 | 7 | 0 | 0 | 6 | 8 | 3 | 54 |
| Kass 2017 | 7 | 4 | | 8 | | 1 | 0 | 6 | 0 | 0 | 0 | 6 | 0 | 0 | 0 | 6 | 8 | 3 | 49 |
| Iijima (2013) | 0 | 4 | | 0 | | 1 | 0 | 6 | 0 | 0 | 0 | 0 | 0 | 0 | 0 | 6 | 8 | 3 | 28 |
| Murphy (2012) | 7 | 4 | | 0 | | 1 | 0 | 6 | 5 | 7 | 8 | 6 | 7 | 8 (T) | 7 (T) | 6 | 8 | 3 | 83 |
| Müller 2019 | 7 | 4 | | 8 | | 1 | 9 | 6 | 5 | 7 | 8 | 6 | 7 | 8 (T) | 7(T) | 6 | 8 | 3 | 100 |
| Noben (2015) | 7 | 4 | | 8 | | 1 | 9 | 6 | 5 | 0 | 8 | 0 | 7 | 8 (T) | 7 (T) | 6 | 8 | 3 | 87 |
| Ride (2016) | 7 | 4 | | 8 | | 1 | 9 | 6 | 5 | 7 | 8 | 6 | 7 | 8 (T) | 7 (T) | 6 | 8 | 3 | 100 |
| Thanh (2013) | 7 | 4 | | 0 | | 1 | 0 | 6 | 5 | 7 | 0 | 6 | 7 | 8 | 7 | 6 | 0 | 3 | 67 |
| Schotanus-Dijkstra, 2018 | 7 | 4 | | 8 | | 0 | 0 | 6 | 5 | 7 | 8 | 6 | 7 | 8 (T) | 7 (T) | 0 | 8 | 0 | 81 |
| Pil (2013) | 7 | 4 | | 0 | | 1 | 9 | 6 | 5 | 7 | 8 | 6 | 7 | 8 | 7 | 6 | 8 | 3 | 92 |
| Atkins (2013) | 7 | 0 | | 0 | | 1 | 0 | 6 | 0 | 0 | 0 | 0 | 0 | 0 | 0 | 0 | 8 | 0 | 22 |
| Lebenbaum (2020) | 7 | 4 | | 8 | | 0 | 9 | 6 | 5 | 7 | 8 | 6 | 7 | 8 | 7 | 6 | 8 | 3 | 99 |
| Damerow (2020) | 7 | 4 | | 0 | | 0 | 9 | 6 | 0 | 7 | 8 | 0 | 0 | 0 | 0 | 6 | 8 | 3 | 58 |
| Dunlap (2019) | 7 | 4 | | 0 | | 0 | 0 | 6 | 5 | 7 | 9 | 6 | 7 | 8 | 7 | 6 | 8 | 3 | 82 |
| Haddock (2019) | 7 | 4 | | 8 | | 0 | 9 | 0 | 5 | 7 | 9 | 6 | 7 | 8 | 7 | 6 | 8 | 3 | 93 |
| Wijnen (2020) | 7 | 4 | | 8 | | 0 | 9 | 6 | 5 | 7 | 9 | 6 | 7 | 8 | 7 | 6 | 8 | 3 | 99 |
| Vasiliadis, (2015) | 7 | 4 | | 0 | | 1 | 9 | 6 | 5 | 7 | 8 | 6 | 7 | 8 (T) | 7 (T) | 0 | 8 | 0 | 83 |
| Denchev, 2018 | 7 | 4 | | 8 | | 0 | 9 | 6 | 5 | 7 | 8 | 6 | 7 | 8 | 7 | 0 | 8 | 0 | 90 |
| Miller (2007) | 7 | 4 | | 0 | | 1 | 0 | 0 | 5 | 7 | 8 | 6 | 7 | 0 | 0 | 0 | 8 | 3 | 56 |
| Smit, 2009 | 7 | 4 | | 8 | | 0 | 9 | 6 | 5 | 7 | 8 | 6 | 7 | 8(T) | 7(T) | 6 | 8 | 0 | 96 |
| Older adults | | | | | | | | | | | | | | | | | | | |
| Bosmans (2014) | 7 | 4 | | 8 | | 1 | 9 | 6 | 5 | 7 | 8 | 6 | 7 | 8 (T) | 7 (T) | 6 | 8 | 3 | 100 |
| van’t Veer-Tazelaar, (2010) | 7 | 4 | | 8 | | 1 | 9 | 6 | 5 | 7 | 8 | 6 | 7 | 8 (T) | 7 (T) | 6 | 8 | 3 | 100 |
| Joling (2013) | 7 | 4 | | 8 | | 1 | 9 | 6 | 5 | 7 | 8 | 6 | 7 | 8 (T) | 7 (T) | 6 | 8 | 3 | 100 |
| Knapp (2013) | 7 | 4 | | 8 | | 1 | 9 | 6 | 5 | 7 | 8 | 6 | 7 | 8 (T) | 7 (T) | 0 | 8 | 3 | 94 |
| Romeo, 2011 | 7 | 4 | | 8 | | 1 | 9 | 6 | 5 | 7 | 8 | 6 | 7 | 8 (T) | 7 (T) | 6 | 8 | 3 | 100 |
| Underwood, 2013 | 7 | 4 | | 8 | | 1 | 9 | 6 | 5 | 7 | 8 | 6 | 7 | 8 (T) | 7 (T) | 6 | 8 | 0 | 98 |
| Clark (2012) | 7 | 0 | | 0 | | 1 | 0 | 6 | 5 | 0 | 0 | 0 | 0 | 8 (T) | 7 (T) | 0 | 8 | 3 | 45 |

T: trial or pre-post study, Q: question
