# Supplementary material for: Cost-effectiveness evidence of mental health prevention and promotion interventions: A systematic review of economic evaluations
Source: PLoS Med. 2021 May 11;18(5):e1003606. doi: 10.1371/journal.pmed.1003606 (PMC8148329; doi:10.1371/journal.pmed.1003606)
Supplement: S3 Table — (DOCX) [file pmed.1003606.s004.docx]

Table S3. Synthesis of findings using the dominance ranking framework

| Cost | Benefit | Study | Mental health conditions | Prevention strategies | Interventions | Implication for decision makers |
| --- | --- | --- | --- | --- | --- | --- |
| **Children and Adolescents** | | | | | |  |
| + | + | Lynch (2019) (1)  USA | Depression | Targeted | Cognitive-behavioral depression prevention  program (CBP)  Usual care | Unclear |
| - | + | Ssegonja (2020) (2) Sweden | Depression | Targeted | Group based cognitive behavioural therapy (GB-CBT) compared with ‘no intervention’ | Favor intervention |
| - (societal, SDQ)  + (societal, QALY)  + (healthcare, SDQ & QALY) | + | Chatterton (2020) (3)  Australia | Internalising problems | Targeted | Screening + Parenting program (Cool Little Kids) versus usual care | Unclear |
| - | + | Nystrand (2020) (4)  Sweden | Externalizing problems | Targeted | Group-based indicated  parenting programs – COPE  Waitlist control | Favor intervention |
| - | + | Nystrand (2020) (4)  Sweden | Externalizing problems | Targeted | group-based indicated  parenting programs - Comet  Waitlist control | Favor intervention |
| - | + | Nystrand (2020) (4)  Sweden | Externalizing problems | Targeted | Group-based indicated  parenting programs – Incredible Years  Waitlist control | Favor intervention |
| - | + | Nystrand (2020) (4)  Sweden | Externalizing problems | Targeted | Group-based indicated  parenting programs – Connect  Waitlist control | Favor intervention |
| - | + | Nystrand (2020) (4)  Sweden | Externalizing problems | Targeted | Indicated parenting interventions - self-help book  Waitlist control | Favor intervention |
| + | - | Anderson (2014) (5)  UK | Depression | Universal | CBT (School based – Resourceful Adolescent Programme) versus Usual Care | Reject intervention |
| + | + | Lee (2016) (6)  Australia | Depression | Universal | Psychological intervention (Universal) versus do nothing | Unclear |
| + | + | Lee (2016) (6)  Australia | Depression | Targeted | Psychological intervention (indicated) versus do nothing | Unclear |
| + | + | Mihalopoulos (2012) (7)  Australia | Depression | Targeted | Psychological Intervention versus Do Nothing | Unclear |
| + | + | Philipsson (2013) (8) Sweden | Depression; Anxiety | Targeted | Physical intervention (dance) versus Usual Care | Unclear |
| - | + | Stallard (2013)(9)  UK | Depression | Targeted | CBT (classroom-based) versus usual curriculum | Favor intervention |
| + | + | Mihalopoulos (2015) (10)  Australia | Anxiety | Targeted | Psycho-educational programme (parent-focused) versus Do Nothing | Unclear |
| + | + | Simon (2012) (11) Netherlands | Anxiety | Targeted | Child-focused CBT versus Non-Intervention | Unclear |
| - | + | Simon (2012) (11) Netherlands | Anxiety | Targeted | Parent-focused CBT versus Non-Intervention | Favor Intervention |
| + | + | Simon (2013) (12)  Netherlands | Anxiety | Targeted | Child-focused CBT, versus Non-Intervention | Unclear |
| + | + | Simon (2013) (12)  Netherlands | Anxiety | Targeted | Parent-focused CBT, versus Non-Intervention | Unclear |
| + | + | Simon (2013) (12)  Netherlands | Anxiety | Targeted | Parent OR child-focused CBT versus Non-Intervention | Unclear |
| + | + | Ahern (2018) (13)  Europe | Suicide | Universal | Universal CBT school based versus Do Nothing | Unclear |
| + | + | Ahern (2018) (13)  Europe | Suicide | Targeted | Indicated school based versus Do Nothing | Unclear |
| + | + | Ahern (2018) (13)  Europe | Suicide | Universal | Screening intervention versus Do Nothing | Unclear |
| -(Mackay)  +(Queensland or Asutralia) | + | Kinchin 2020 (14)  Australia | Suicide | Universal | SafeTALK (3-hour education session)  Status quo | Unclear |
| - | + | Godoy Garraza (2018) (15)  USA | Suicide | Targeted | Multicomponent program (The GLS Suicide Prevention Program) versus do nothing | Favor intervention |
| - | + | Gray (2011) (16)  USA | Suicide | Targeted | Best practice: early mental health intervention vs control group | Favor intervention |
| + | + | Le (2017) (17)  Australia | Anorexia Nervosa and Bulimia Nervosa | Targeted | Cognitive Dissonance (school-based) versus Do nothing | Unclear |
| + | + | Wang (2011) (18)  USA | Bulimia Nervosa | Universal | School-based education + physical activity versus Usual curricula | Unclear |
| + | + | Beckman (2015) (19)  Sweden | Bullying | Universal | Whole-school approach | Unclear |
| +(Sweden)  -(US) | + | Deogan (2015) (20)  Sweden | Cannabis Use | Universal | ALERT (Adolescent, learning, Experiences, Resistance, and Training) plus ATOD (Alcohol, Tobacco, and Other Drug) versus ordinary ATOD only | Unclear |
| + | + | Ocasio (2014) (21)  USA | Challenging Bahaviour | Universal | Tiered approach: Second Step curriculum, mental health clinician & play therapy. | Unclear |
| + | + | Dalziel (2015) (22)  Australia | Maltreatment | Targeted | Parents under Pressure (PuP) programme vs ‘Usual Care’ and ‘Brief Intervention’ groups | Unclear |
| - | + | Herman (2015) (23)  USA | Mental Health (in women and their children) | Targeted | Parenting-focused program  Parenting-focused program with child focus  No intervention | Favor intervention |
| **Adults** | | | | | |  |
| - | + | Kumar (2018) (24)  USA | Anxiety | Universal | Mobile CBT  no CBT | Favor intervention |
| - | + | Kumar (2018) (24)  USA | Anxiety | Universal | Traditional face-to-face CBT  no CBT | Favor intervention |
| + | + | Lintvedt 2013  Norway (25) | Depression | Unclear | CBT vs. no intervention | Unclear |
| + | + | Dukhovny, 2013  Canada (26) | Depression | Targeted | Peer support vs usual care | Unclear |
| - | + | Henderson, (2019) (27)  UK | Depression | Universal | PoNDER health visitor training (intervention cluster)  Control cluster | Favor intervention |
| - | + | Lokkerbol 2014  Netherlands (28) | Depression | Universal | Self-help e-heath+usual care vs usual care | Favor intervention |
| + | + | Mihalopoulos 2011  Australia (29) | Depression | Targeted | CBT vs. do nothing | Unclear |
| + | + | Mihalopoulos 2011  Australia (29) | Depression | Targeted | Brief bibliotherapy vs do nothing | Unclear |
| + | + | Buntrock et al. 2017 (30)  Germany | Depression | Targeted | iPST/BA + TAU vs enhanced TAU | Unclear |
| - | + | Hunter et al. 2014 (31)  UK | Depression | Targeted | Screening + low intensity preventive intervention vs. Universal preventive intervention | Favor intervention |
| + | + | Hunter et al. 2014 (31)  UK | Depression | Targeted | Screening + low intensity preventive intervention vs. treatment as usual | Unclear |
| -(societal perspective)  +(health sector perspective) | + | Van den Berg 2011 (32)  Netherlands | Depression | Targeted | Screening + psychotherapy vs usual care | Unclear |
| -(societal perspective)  +(health sector perspective) | + | Fernandez et al. 2018 (33)  Spain | Depression | Targeted | Screening + low intensity preventive intervention vs. treatment as usual | Unclear |
| + | + | Jiao 2017 (34)  USA | Depression | Universal | Screening + treatment vs. no screening | Unclear |
| - | + | Goetzel 2014 (35)  US | Modifiable risk factors including depression | Universal | Health risk management program vs no intervention | Favor intervention |
| - | + | Ising et al. 2015, 2017 (36, 37)  Netherlands | Psychosis | Targeted | CBT + TAU vs. TAU | Favor intervention |
| - | + | Wijnen 2020 (38)  Netherlands | Psychosis | Targeted | CBT  TAU | Favor intervention |
| + | + | Akers et al. 2017 (39)  USA | Eating Disorders | Targeted | Cognitive dissonance vs. educational brochure | Unclear |
| - | + | Kass 2017 (40)  USA | Eating Disorders | Universal | Screening + preventive or treatment vs. waitlist | Favor intervention |
| - | + | Iijima 2013 (41)  Japan | Mental health | Universal | Mental health prevention vs. no intervention | Favor intervention |
| + | + | Murphy 2012 (42)  UK | Mental health | Targeted | National Exercise referral scheme vs. Usual care | Unclear |
| - (societal)  + (healthcare) | + | Müller, (2019) (43)  Germany | Mental health | Universal | Mindfulness-based mental health promotion program Life Balance  Usual care | Unclear |
| - | + | Noben 2014 2015 (44, 45)  Netherlands | Mental health | Targeted | Screen + personalized feedback + occupational physician  Screen + without feedback + usual care | Favor intervention |
| + | + | Ride 2016 (46)  Australia | Mental health | Targeted | Psychoeducational  Usual care | Unclear |
| - | + | Thanh 2013 (47)  Canada | Mental health | Targeted | Service network  No service network | Favor intervention |
| + | + | Schotanus-Dijkstra, 2018 (48), Netherland | Mental health | Targeted | Guided positive psychology  Waistlist | Unclear |
| - | + | Pil 2013 (49)  Belgium | Suicide | Targeted | Suicide Helpline  No intervention | Favor intervention |
| +  -(ROI) | + | Atkins 2013 (50)  USA | Suicide | Universal | Barrier on the Golden Gate Bridge  No intervention | Unclear |
| + | + | Lebenbaum 2020 (51)  Canada | Suicide | Universal | Multicomponent  No intervention | Unclear |
| + | + | Damerow 2020 (52)  Sri Lanka | Suicide | Universal | Shop-based gatekeeper training program | Unclear |
| + | + | Dunlap 2019 (53)  USA | Suicide or suicide attempt | Targeted | Universal screening  Universal screening + telephone  Usual care | Unclear |
| - | - | Haddock 2019 (54)  UK | Suicide | Targeted | CBT + TAU  TAU | Unclear |
| + | + | Vasiliadis 2015 (55), Canada | Suicide | Targeted | NAD Multi modal suicidal prevention program | Unclear |
| - | + | Denchev, 2018 (56) USA | Suicide | Targeted | Postcard vs usual care | Favor intervention |
| + | + | Denchev, 2018 (56) USA | Suicide | Targeted | Telephone outreach vs usual care | Unclear |
| + | + | Denchev, 2018 (56) USA | Suicide | Targeted | CBT vs usual care | Unclear |
| - | + | Miller (2007) (57), USA | Substance abuse | Universal | Peer-based substance abuse prevention | Favor intervention |
| + | + | Smit, 2009 (58) Netherland | Panic disorders | Targeted | CBT vs usual care | Unclear |
| **Older adults** | | | | | |  |
| + | + (QALYs)  + (incidence depression or anxiety)  - (incidence depression)  + (incidence anxiety)  - (improvement CES-D or HADS-A) | Bosmans (2014) (59)  Netherlands | Depression & Anxiety | Targeted | Stepped care programme vs. Usual care | Unclear |
| + | + | van’t Veer-Tazelaar, (2010),(60) Netherlands | Depression and Anxiety | Targeted | Stepped care preventive intervention vs. routine primary care | Unclear |
| + | + (dyad QALY)  - (caregiver QALY)  + (patient QALY)  - (caregiver incident of depression and/or anxiety) | Joling (2013) (61)  Netherlands | Depression and Anxiety | Targeted | Family meetings intervention vs. Usual care | Unclear |
| + | + | Knapp  (2013),(62)  UK | Depression and Anxiety | Targeted | Eight session, manual based, coping intervention vs. usual care | Unclear |
| + | + | Romeo (2011),(63)  UK | Depression | Targeted | CBT vs. Treatment as usual | Unclear |
| + | - | Underwood (2013), (64)  UK | Depression | Targeted | Physical intervention (Whole-home intervention) compared to control home | Reject intervention |
| + | + | Clark (2012), (37)  USA | Mental wellbeing | Universal | Occupational therapy intervention (Well Elderly Lifestyle Redesign intervention) vs. usual care | Unclear |

CBT: Cognitive behavioral therapy; TAU: treatment as usual, ATOD: Alcohol, Tobacco, and Other Drug; iPST: internet problem solving therapy

USA: the United States of America; UK: The United Kingdom
